# Hippocampal input-driven plasticity of prefrontal interneurons reveals a circuit basis for impaired spatial working memory

**DOI:** 10.1101/2025.07.21.665987

**Published:** 2025-07-24

**Authors:** Shana E. Silverstein, Thomas T. Clarity, Meena S. Deshpande, Erik Vaughan, Shoshana Novik, Hector E. Yarur, Shiliang Zhang, Valerie S. Tsai, Rong Ye, Rachel M. Mikofsky, Madeline Hsiang, Avery Bauman, Gabriel Loewinger, Francisco Pereira, Marisela Morales, Vikaas S. Sohal, Hugo A. Tejeda, Joshua A. Gordon, David A. Kupferschmidt

**Affiliations:** 1 Integrative Neuroscience Section, National Institute of Neurological Disorders and Stroke, National Institutes of Health, Bethesda, MD, USA; 2 Unit on Neuromodulation and Synaptic Integration, National Institute of Mental Health, National Institutes of Health, Bethesda, MD, USA; 3 Confocal and Electron Microscopy Core, National Institute on Drug Abuse, National Institutes of Health, Baltimore, MD, USA; 4 Department of Neuroscience, Brown University, Providence, RI, USA; 5 Department of Psychiatry, Vagelos College of Physicians and Surgeons, Columbia University, New York, NY, USA; 6 Machine Learning Team, National Institute of Mental Health, National Institutes of Health, Bethesda, MD, USA; 7 Integrative Neuroscience Research Branch, National Institute on Drug Abuse, National Institutes of Health, Baltimore, MD, USA; 8 Department of Psychiatry and Behavioral Sciences and Weill Institute for Neurosciences, Center for Integrative Neuroscience and Kavli Institute for Fundamental Neuroscience, University of California, San Francisco, San Francisco, CA, USA; 9 Office of the Director, National Institute of Mental Health, National Institutes of Health, Rockville, MD, USA; 10 Office of the Director, New York State Psychiatric Institute, New York, NY, USA; 11 Lead contact

## Abstract

Dynamic functional connectivity between the ventral hippocampus (vHPC) and medial prefrontal cortex (mPFC) is essential for spatial working memory (SWM). Interactions between vHPC projections and mPFC interneurons, and their plasticity, are uniquely positioned to influence SWM, yet the nature of these interactions remains unclear. Here, we combined *in vivo* optical stimulation of vHPC inputs to mPFC with calcium recordings of discrete mPFC interneuron populations in mice, revealing class-specific response profiles and plasticity. Repeated vHPC input stimulation persistently depressed activity in vasoactive intestinal peptide (VIP)-expressing interneurons and potentiated activity in somatostatin-expressing interneurons. *Ex vivo* whole-cell electrophysiology and computational modeling revealed that these divergent effects likely arise from a primary weakening of monosynaptic vHPC input onto VIP interneurons. Leveraging this plasticity to inform the circuit interactions that support SWM, we found that mice with prior vHPC input stimulation displayed elevated VIP interneuron activity during the delay epoch in early SWM task training, and this enhanced activity correlated with poorer training performance. Accordingly, mice modeling the schizophrenia-predisposing 22q11.2 deletion syndrome with known SWM learning deficits recapitulated this aberrant VIP interneuron activity profile and showed reduced vHPC targeting of mPFC VIP interneurons. Together, these findings reveal novel cell-type-specific plasticity in cognition-supporting circuits and illustrate how reweighting of inputs to VIP interneurons may contribute to working memory dysfunction.

## IN BRIEF

Silverstein et al. investigated how ventral hippocampus (vHPC) inputs to medial prefrontal cortex (mPFC) interact with mPFC interneuron populations to support spatial working memory (SWM) in mice. They show that repeated vHPC input stimulation that reduces vHPC drive onto vasoactive intestinal peptide (VIP) interneurons also elevates delay-related VIP interneuron activity in early SWM task training, and this enhanced activity correlates with poorer training performance. Mice modelling the 22q11.2 deletion syndrome—known to have SWM learning deficits—recapitulate this altered VIP interneuron activity and show reduced vHPC input targeting of VIP interneurons.

## GRAPHICAL ABSTRACT

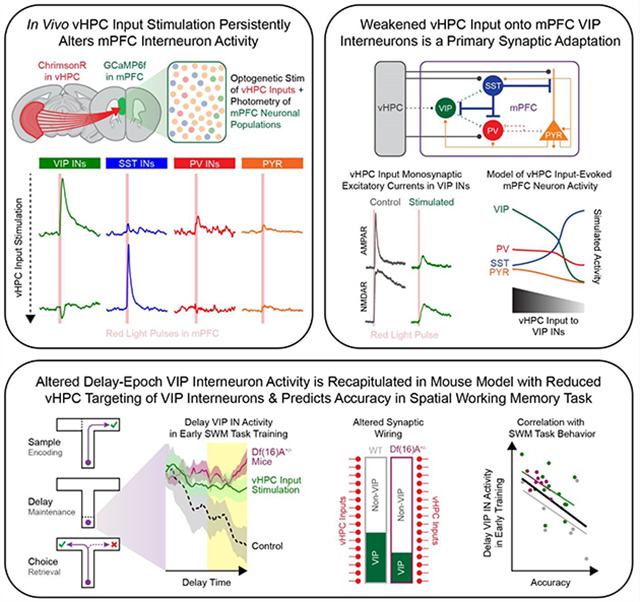

## INTRODUCTION

Functional interactions between the hippocampus (HPC) and prefrontal cortex (PFC) are essential for various learning and memory processes ^1–3^. Spatial working memory (SWM)—the ability to encode, maintain, and retrieve spatial information over short timescales—relies on interacting circuits within and between the HPC and PFC ^4–6^. HPC-PFC interactions are reshaped by spatial learning, including acquisition of a SWM task ^7–10^. In turn, manipulations that modify these interactions (e.g., activity-induced synaptic plasticity) can influence spatial learning ^11–14^. Altered HPC-PFC circuit connectivity has also been linked to psychiatric disease-related impairments in SWM and other cognitive functions in humans and animal models ^15–19^.

Direct neuronal projections from the rodent ventral HPC (vHPC) to the medial PFC (mPFC) mediate SWM behavior and associated HPC-mPFC functional connectivity ^20–22^. Inhibition of these monosynaptic vHPC inputs during the “sample” (encoding) epoch of a SWM task impairs task performance, encoding of task-relevant spatial information in mPFC, and vHPC-mPFC oscillatory synchrony ^6,22^. These and other findings, including from tasks of spatial approach-avoidance decision making ^23^, suggest that vHPC inputs dynamically coordinate mPFC neuronal populations to convey task-relevant spatial information necessary for normal spatial cognition. vHPC inputs to mPFC can also drive persistent synaptic plasticity capable of altering spatial cognition ^24,25^. Indeed, sustained optogenetic stimulation of vHPC inputs to mPFC strengthened functional connectivity between the two regions and impaired the ability of mice to overcome pre-established navigation biases to learn a SWM task ^14^.

Discrete classes of inhibitory mPFC interneurons known to receive monosynaptic excitatory input from vHPC ^26–28^ also support SWM and vHPC-mPFC interactions ^29,30^. One such class, vasoactive intestinal polypeptide (VIP)-expressing interneurons, principally target other interneuron classes to disinhibit pyramidal neurons ^31–33^. mPFC VIP interneurons contribute to SWM maintenance, showing robust activity across the delay epoch of SWM tasks that represent information about prior sample location ^34^. Inhibiting these interneurons, both generally and specifically in the delay epoch, disrupts performance in spatial and non-spatial WM tasks ^34,35^. In turn, activating VIP interneurons in the delay epoch improves performance in previously learned delayed-response tasks and amplifies representations of task-relevant information in the activity of VIP interneurons and pyramidal neurons ^34,35^. Although direct evidence of cooperation between VIP interneurons and vHPC inputs in SWM is lacking, activity of VIP interneurons—like vHPC inputs ^23,36^—supports mPFC neuronal representations of task-relevant spatial information to guide approach-avoidance decision making, and does so preferentially when vHPC-mPFC theta-frequency synchrony is strong ^37^. Another interneuron class, somatostatin (SST)-expressing interneurons, show strong reciprocal inhibitory connections with VIP interneurons and principally target distal dendrites of pyramidal neurons to gate influence of their excitatory inputs ^38–40^. Paralleling the effects of vHPC input inhibition, SST interneuron inhibition during the sample epoch of a SWM task impairs task performance, encoding of task-relevant spatial information in mPFC neuronal activity, and vHPC-mPFC synchrony ^41^. These studies suggest that vHPC inputs and SST interneurons may also cooperate to support SWM encoding.

Altogether, these findings indicate that plastic interactions between vHPC inputs and select mPFC interneuron classes are well-positioned to exert dynamic control over SWM. Direct study of the nature and behavioral contributions of these interactions, however, is lacking. Here, by combining optical stimulation of vHPC inputs and recordings of mPFC VIP, SST, and parvalbumin (PV)-expressing interneuron activity, we characterized *in vivo* functional connectivity and plasticity between vHPC inputs and mPFC interneurons. We then leveraged this plasticity—and the activity and connectivity of interneurons in a disease-relevant mouse model with impaired SWM task learning—to inform how vHPC inputs and mPFC interneurons interact to support SWM. We hypothesized that weakening functional connectivity between vHPC inputs and mPFC interneurons would impair SWM task learning. We found that repeated vHPC input stimulation depressed evoked and spontaneous *in vivo* activity of VIP interneurons while enhancing that of SST interneurons, consistent with their strong reciprocal inhibitory connectivity. Using slice electrophysiology and computational modeling, we identified weakened monosynaptic vHPC input onto VIP interneurons as a primary driver of these population-level changes. Despite having minimal impact on overall SWM behavior, prior vHPC input stimulation enhanced delay-epoch VIP interneuron activity in early SWM task training, and this heightened activity correlated with poorer training performance. Finally, we show that VIP interneurons in *Df(16)A*^+/−^ mice—a model of the 22q11.2 deletion syndrome with known SWM learning deficits^18,42^—recapitulate the heightened delay activity seen in VIP interneurons of stimulated mice and receive proportionally fewer monosynaptic inputs from vHPC. These data reveal cell-type-specific plasticity of vHPC inputs onto mPFC interneurons and highlight how reweighting of VIP interneuron inputs—whether by neural activity or genetic mutation—may contribute to working memory dysfunction.

## RESULTS

### Repeated stimulation of vHPC inputs to mPFC differentially alters activity of specific mPFC neuronal populations

**Figure 1.**
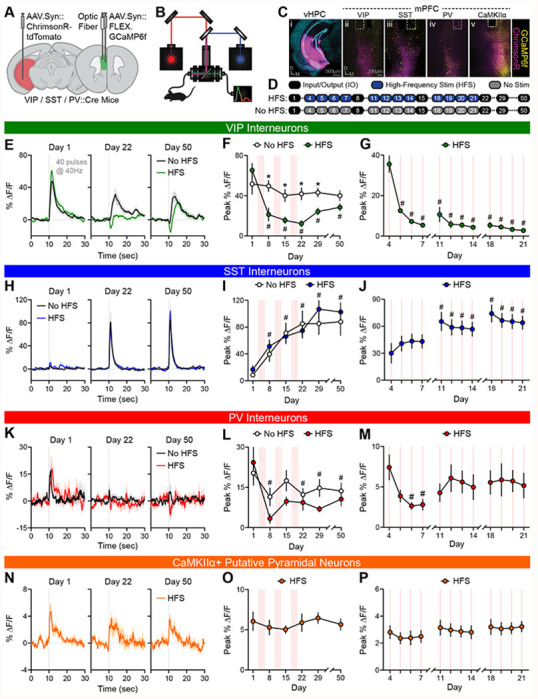
Repeated *in vivo* vHPC input stimulation differentially alters evoked activity of specific mPFC neuronal populations. (**A**) Schematic showing AAV.Syn::ChrimsonR-tdTomato in unilateral vHPC, AAV.Syn::FLEX.GCaMP6f in unilateral mPFC, and an optic fiber in unilateral mPFC of VIP, SST, or PV::Cre mice. (**B**) Schematic of optogenetic stimulation-compatible, spectral-based photometry system. (**C**) Representative viral ChrimsonR-tdTomato expression (pink) in vHPC (i) and vHPC terminals in mPFC (ii-v), GCaMP6f expression (yellow) in mPFC interneurons (VIP [ii], SST [iii], or PV [iv]) or CaMKIIα-expressing neurons (v; achieved using AAV.CaMKIIα::GCaMP6f in C57BL/6J mice). Optic fiber placement denoted by dashed lines. DAPI staining (cyan) shown in vHPC image. (**D**) Schematic of 50-day experimental protocol for HFS and No HFS mice. (**E**) Average stimulation-evoked Ca^2+^ responses of mPFC VIP interneurons in No HFS and HFS mice on IO Days 1, 22, and 50. Stimulation (40 pulses at 40 Hz) initiated at 10-sec timepoint. (**F**) Average peak stimulation-evoked Ca^2+^ responses of VIP interneurons in No HFS and HFS mice across all six IO days. Stimulation was 40 pulses at 40 Hz. 2-way ANOVA, Main effect of Day: F(3.349, 70.33)=12.81, p<0.001; Main effect of Stimulation: F(1, 21)=7.903, p<0.05; Day x Stimulation interaction: F(5, 105)=6.416, p<0.0001; *p<0.005, different from HFS mice; #p<0.005 different from Day 1; n=11,12. (**G**) Average peak stimulation-evoked Ca^2+^ responses of VIP interneurons in HFS mice across HFS test days. RM ANOVA, Main effect of Day: F(2.498, 27.47)=14.56, p<0.0001; #p<0.05, different from Day 4; n=12. (**H**) Average stimulation-evoked Ca^2+^ responses of mPFC SST interneurons in No HFS and HFS mice on IO Days 1, 22, and 50. Stimulation (40 pulses at 40 Hz) initiated at 10-sec timepoint. (**I**) Average peak stimulation-evoked Ca^2+^ responses of SST interneurons in No HFS and HFS mice across all six IO days. Stimulation was 40 pulses at 40 Hz. 2-way ANOVA, Main effect of Day: F(1.934, 44.49)=29.48, p<0.0001; #p<0.001, different from Day 1; n=12,13. (**J**) Average peak stimulation-evoked Ca^2+^ responses of SST interneurons in HFS mice across HFS test days. RM ANOVA, Main effect of Day: F(1.998, 21.97)=16.88, p<0.0001; #p<0.05, different from Day 4; n=12. (**K**) Average stimulation-evoked Ca^2+^ responses of mPFC PV interneurons in No HFS and HFS mice on IO Days 1, 22, and 50. Stimulation (40 pulses at 40 Hz) initiated at 10-sec timepoint. (**L**) Average peak stimulation-evoked Ca^2+^ responses of PV interneurons in No HFS and HFS mice across all six IO days. Stimulation was 40 pulses at 40 Hz. Mixed-effects model, Main effect of Day: F(2.670, 36.85)=9.961, p≤0.0001; #p<0.05, different from Day 1; n=7,9. (**M**) Average peak stimulation-evoked Ca^2+^ responses of PV interneurons in HFS mice across HFS test days. RM ANOVA, Main effect of Day: F(3.569, 32.12)=3.589, p<0.05; #p<0.05, different from Day 4; n=10. (**N**) Average stimulation-evoked Ca^2+^ responses of mPFC CaMKIIα+ putative pyramidal neurons in HFS mice on IO Days 1, 22, and 50. Stimulation (40 pulses at 40 Hz) initiated at 10-sec timepoint. (**O**) Average peak stimulation-evoked Ca^2+^ responses of CaMKIIα+ neurons in HFS mice across all six IO days; n=10. Stimulation was 40 pulses at 40 Hz. (**P**) Average peak stimulation-evoked Ca^2+^ responses of CaMKIIα+ neurons in HFS mice across HFS test days. RM ANOVA, Main effect of Day: F(3.626, 32.63)=3.730, p<0.05; n=10.

*In vivo* functional interactions between mouse vHPC inputs and mPFC neurons were characterized using an all-optical approach. The red-shifted opsin ChrimsonR was expressed in vHPC projection neurons and the green fluorescent Ca^2+^ indicator GCaMP6f in one of several mPFC neuronal subpopulations. Simultaneously, vHPC terminals in mPFC were stimulated with red light and mPFC neuron Ca^2+^ activity was measured by fiber photometry through the same fiber in mPFC (Figure 1A–B; S1). Stimulation-evoked responses were recorded from interneurons expressing VIP, SST, or PV, or putative pyramidal neurons expressing Ca^2+^/calmodulin-dependent protein kinase IIα (CaMKIIα; Figure 1C).

Over 50 days, mice underwent six input-output (IO) sessions to systematically characterize mPFC neuron responses to vHPC input stimulation across time (Figure 1D). During each IO session, vHPC terminals were stimulated using a range of pulse frequencies and numbers (two rounds of 18 trains ranging from 1-40 pulses at 5, 10, 20, 30, and 40 Hz) while recording Ca^2+^ responses from each neuronal population (Figure S1C). A separate cohort of mice underwent additional high-frequency stimulation (HFS) consisting of 100 daily trains of 40 pulses delivered at 40 Hz over 12 days early in the experimental timeline. The additional HFS was designed to induce potential plasticity in the IO Ca^2+^ responses (Figure 1D).

VIP interneuron responses on the first IO day scaled with increasing stimulation pulse numbers and frequencies (Figure S2A). Responses to the strongest IO stimulation (40 pulses at 40 Hz) were initially pronounced and subtly decreased across IO days in mice receiving only IO stimulation (No HFS mice; Figure 1E,F; S2A; S3A–D). Modest decreases in peak amplitude across IO days (Figure 1E,F; S2A) were accompanied by significant response reductions during the stimulation window and increased latency to reach peak responses (Figure S3A–D). In HFS mice, VIP interneuron responses to IO stimulation were rapidly diminished by the additional HFS. This response depression persisted across subsequent IO sessions with some recovery in the weeks following the final HFS (Figure 1E,F). Importantly, the depression of VIP interneuron responses was evident when probed with various pulse numbers and frequencies (Figure S2A). Moreover, VIP interneuron responses to the HFS itself markedly diminished across the first few HFS sessions (Figure 1G) and within the first HFS session (Figure S2C).

In contrast to VIP interneuron responses, SST interneuron Ca^2+^ responses to vHPC input stimulation on the initial IO day were small but reliably detectable (Figure 1H,I; S2B). Unexpectedly, in mice that received IO stimulation only (No HFS group), SST interneuron responses gradually and dramatically enhanced over 50 days (Figure 1H,I). This response potentiation persisted across a three-week period without stimulation (Days 29 and 50) and was not restricted to a particular set of pulse numbers and frequencies (Figure S2B). Interestingly, the potentiation of SST interneuron responses seen in No HFS mice was indistinguishable from HFS mice, indicating that additional HFS did not further potentiate SST interneuron responses (Figure 1H,I). SST interneuron responses to the HFS itself gradually enhanced over consecutive HFS sessions (Figure 1J). Once potentiated, responses to HFS showed modest depression within a single session (Figure S2C).

Given that SST interneuron potentiation was indistinguishable in No HFS and HFS mice, a separate experiment was conducted to dissociate the impact of stimulation from other non-specific factors, including changes in viral expression, mouse handling, or habituation. Mice either received IO stimulation on Days 1, 8, 15 and 22 or were treated otherwise identically but received their first IO stimulation on Day 22. SST interneuron responses on Day 22 were significantly lower in the group receiving Day 22 stimulation only than those that received four IO stimulation sessions (Figure S3E–G). Moreover, responses on the initial IO stimulation days were indistinguishable between groups (Figure S3H). These findings confirmed that the stimulation comprising the IO sessions alone is sufficient to potentiate SST interneuron responses. Similarly, HFS alone (without interleaved IO sessions, assessed in a subsequent experiment) produced the same degree of SST response potentiation (Figure 5C,E).

Like SST interneuron responses, PV interneuron responses to initial IO stimulation were small. However, they depressed comparably in mice with or without additional HFS over subsequent stimulation sessions (Figure 1K–M; Figure S2C). In contrast to the highly plastic interneuron responses, responses in CaMKIIα-expressing putative pyramidal neurons were relatively small and stable throughout the 50-day experiment (Figure 1N–P; Figure S2C).

**Figure 2.**
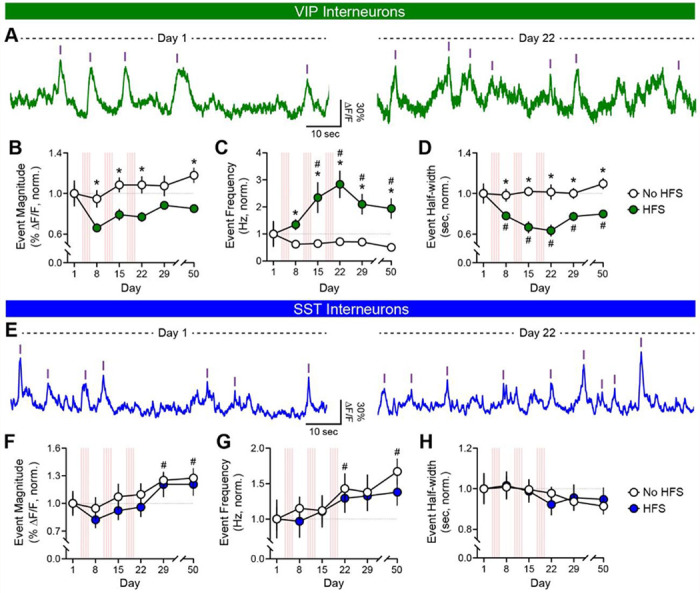
Repeated vHPC input stimulation alters spontaneous Ca^2+^ dynamics in VIP and SST interneurons. (**A**) Spontaneous VIP interneuron Ca^2+^ events during baseline periods on IO Days 1 and 22. (**B**) Normalized average event magnitude of spontaneous Ca^2+^ events of mPFC VIP interneurons in No HFS and HFS mice across six IO days. 2-way ANOVA, Main effect of Day: F(2.539, 49.27)=4.588, p<0.01; Main effect of Stimulation: F(1, 19)=6.935, p<0.05; Day x Stimulation interaction: F(5, 95)=3.066, p<0.05; *p<0.01 different from HFS; n=10,11. (**C**) Event frequency of normalized average spontaneous Ca^2+^ events of mPFC VIP interneurons in No HFS and HFS mice across six IO days. 2-way ANOVA, Main effect of Day: F(2.690, 51.11)=4.168, p<0.05; Main effect of Stimulation: F(1, 19)=11.02, p<0.005; Day x Stimulation interaction: F(5, 95)=6.309, p<0.0001; *p<0.05 different from HFS; #p<0.05 HFS different from HFS Day 1. (**D**) Event half-width of normalized average spontaneous Ca^2+^ events of mPFC VIP interneurons in No HFS and HFS mice across six IO days. 2-way ANOVA, Main effect of Day: F(2.195, 41.70)=3.625, p<0.05; Main effect of Stimulation: F(1, 19)=18.14, p<0.0005; Day x Stimulation interaction: F(5, 95)=3.942, p<0.005; *p<0.05 different from HFS; #p<0.05 HFS different from HFS Day 1. (**E**) Spontaneous SST interneuron Ca^2+^ events during 10-min baseline periods on IO Days 1 and 22. (**F**) Normalized average event magnitude of spontaneous Ca^2+^ events of mPFC SST interneurons in No HFS and HFS mice during baseline periods of the six IO days. 2-way ANOVA, Main effect of Day: F(2.915, 40.80)=9.144, p<0.0001; #p<0.005 different from Day 1; n=7-9. (**G**) Normalized average event frequency of spontaneous Ca^2+^ events of mPFC SST interneurons in No HFS and HFS mice during baseline periods of the six IO days. 2-way ANOVA, Main effect of Day: F(2.444, 34.22)=7.094, p<0.05; #p<0.05 different from Day 1. (**H**) Normalized average event half-width of spontaneous Ca^2+^ events of mPFC SST interneurons in No HFS and HFS mice during baseline periods of the six IO days.

To assess whether vHPC input stimulation modulated spontaneous activity profiles of mPFC interneurons—beyond their stimulation-evoked responses—endogenous Ca^2+^ transients of VIP and SST interneurons during the 10-min baseline periods prior to each IO session were analyzed. Consistent with the plasticity observed in evoked VIP interneuron responses, spontaneous VIP interneuron Ca^2+^ transients were depressed by HFS, showing reductions in magnitude and half-width in HFS relative to No HFS mice (Figure 2A–D); unexpectedly, however, transient frequency was increased by HFS. In contrast, spontaneous SST interneuron Ca^2+^ transients increased in magnitude and frequency across weekly IO sessions in both No HFS and HFS mice (Figure 2E–H). Importantly, No HFS and HFS mice showed indistinguishable behavior (e.g., locomotion, time spent in center of chamber) during these baseline periods (data not shown). Together, these findings reveal that vHPC input stimulation induces striking cell-type-specific plasticity in the spontaneous activity of mPFC interneurons and in their *in vivo* functional connectivity with vHPC inputs.

### Prior vHPC input stimulation reduces vHPC monosynaptic connectivity with mPFC VIP interneurons

**Figure 3.**
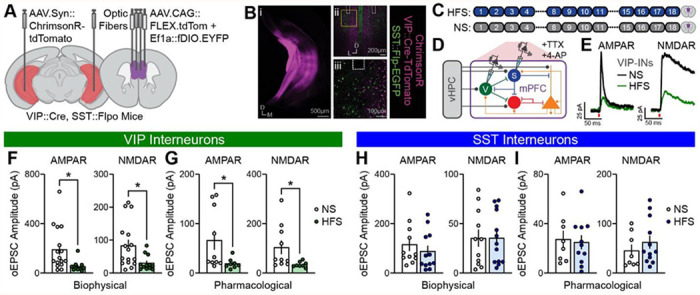
Prior vHPC input stimulation reduces vHPC monosynaptic connectivity with mPFC VIP interneurons. (**A**) Schematic showing AAV.Syn::ChrimsonR-tdTomato in bilateral vHPC, AAV:CAG::FLEX.tdTomato and AAV:Ef1a::fDIO.EYFP in bilateral mPFC, and optic fibers in bilateral mPFC of VIP::Cre, SST::Flpo mice. (**B**) Representative viral ChrimsonR-tdTomato expression (magenta) in vHPC (i) and vHPC terminals in mPFC (ii-iii), tdTomato expression (magenta) in VIP interneurons and EGFP expression (green) in SST interneurons (ii-iii). Optic fiber placement denoted by dashed lines. (**C**) Schematic of 18-day experimental protocol consisting of either 12 HFS sessions or no stimulation (NS). Euthanasia and recordings were conducted 24 hours following final stimulation. (**D**) Schematic of brain slice whole-cell electrophysiology recording of monosynaptic AMPA receptor (AMPAR)- and NMDA receptor (NMDAR)-mediated currents in VIP or SST interneurons evoked by optogenetic stimulation of vHPC inputs to mPFC. (**E**) Representative vHPC input stimulation-evoked monosynaptic AMPAR- and NMDAR-mediated currents (pharmacologically isolated) in VIP interneurons of a NS or HFS mouse. (**F**) Average biophysically isolated AMPAR- and NMDAR-mediated monosynaptic oEPSC amplitude in VIP interneurons of NS and HFS mice. AMPAR: *p<0.005, U=33; NMDAR: *p<0.01, U=40; Mann Whitney tests; n=16,12 cells. (**G**) Average pharmacologically isolated AMPAR- and NMDAR-mediated monosynaptic oEPSC amplitude in VIP interneurons of NS and HFS mice. Mann-Whitney tests, AMPAR: *p<0.05, U=15; NMDAR: *p<0.01, U=11; n=10,8 cells. (**H**) Average biophysically isolated AMPAR- and NMDAR-mediated optically evoked monosynaptic oEPSC amplitude in SST interneurons of NS and HFS mice; n=11-13 cells. (**I**) Average pharmacologically isolated AMPAR- and NMDAR-mediated monosynaptic oEPSC amplitude in SST interneurons of NS and HFS mice; n=8-12 cells.

To characterize potential synaptic adaptations underlying the plasticity observed *in vivo*, vHPC monosynaptic connectivity with mPFC VIP or SST interneurons was assessed using whole-cell electrophysiology in mPFC tissue from mice with or without prior vHPC input stimulation. Repeated HFS—without accompanying IO stimulation—was used given that it induces robust plasticity in both interneuron populations. Mice expressing ChrimsonR in vHPC and tdTomato and EGFP in mPFC VIP and SST interneuron populations, respectively, received either 12 HFS sessions or No Stimulation (NS) (Figure 3A–C). One day following the final stimulation, brain slices containing mPFC were prepared and whole-cell voltage-clamp recordings of optogenetically evoked excitatory postsynaptic currents (oEPSCs) were made from identified VIP and SST interneurons. Slices were pretreated with Na^+^ channel blocker tetrodotoxin (TTX) and K^+^ channel blocker 4-aminopyridine (4-AP) to isolate monosynaptic currents induced by red light activation (1-ms pulses, 10-sec inter-pulse interval) of ChrimsonR-expressing vHPC inputs ^43^ (Figure 3D). AMPA receptor (AMPAR)- and NMDA receptor (NMDAR)-mediated oEPSCs were isolated biophysically (holding cells at −70 and +40 mV, respectively) and pharmacologically (+40 mV holding, in presence of D-AP5 or after subtraction of D-AP5-isolated currents, respectively; Figure 3E; S4A,C). In VIP interneurons, amplitudes of both AMPAR- and NMDAR-mediated oEPSCs were robustly reduced in mice with prior vHPC input HFS (Figure 3F,G), with no change in their ratio (Figure S4B). In contrast, AMPAR- and NMDAR-mediated oEPSC amplitudes (Figure 3H,I) and ratios (Figure S4D) in SST interneurons obtained from the same animals were unaltered by prior HFS relative to recordings from NS mice. These findings indicate that repeated *in vivo* vHPC input stimulation weakens vHPC monosynaptic excitatory input to mPFC VIP but not SST interneurons.

### Computational model shows that weakening vHPC input onto VIP interneurons can plausibly enhance SST interneuron responses and reduce PV interneuron responses

**Figure 4.**
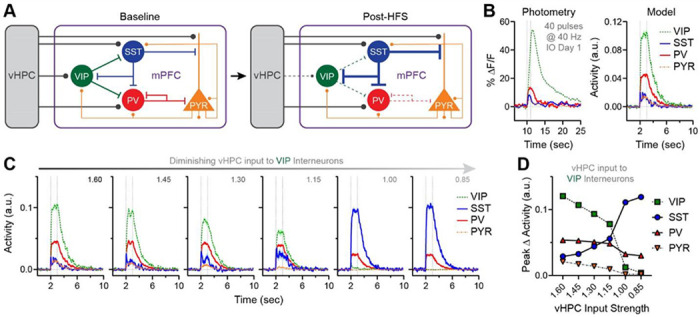
Computational model shows that weakened vHPC input onto VIP interneurons can plausibly enhance SST interneuron responses and reduce PV interneuron responses. (**A**) Conceptual model of hypothesized changes in mPFC microcircuit following vHPC input HFS. (**B**) Comparison of Ca^2+^ activity (left) and computationally modeled activity (right) in response to real or modeled vHPC input stimulation in mPFC neuronal populations or their model analogs. Left: Average stimulation-evoked Ca^2+^ responses of mPFC neuronal populations on IO Day 1. Stimulation (40 pulses at 40 Hz) initiated at 10-sec timepoint. Right: Simulated activity from integrate-and-fire computational model of mPFC microcircuit evoked by stimulation of “vHPC” input. In the model, stimulation (1 sec) initiated at 2-sec timepoint. (**C-D**) Simulated traces (**C**) and peak activity (**D**) for each neuronal population during responses to vHPC input stimulation, shown for diminishing strengths of vHPC input to VIP interneurons (values ranging from 1.6 [initial] to 0.85).

Collectively, these *in vivo* and *ex vivo* findings support a conceptual model (Figure 4A) whereby blunting the monosynaptic drive of vHPC inputs onto VIP interneurons may reduce VIP interneuron responses and disinhibit SST interneuron responses to vHPC input stimulation. This heightened evoked SST interneuron activity may suppress PV interneuron responses and further suppress VIP interneuron responses. Pyramidal neurons may receive a net balance of increased inhibition from SST interneurons and decreased inhibition from PV interneurons, yielding relatively stable responses to repeated vHPC input stimulation.

To test whether weakening of monosynaptic input to VIP interneurons could plausibly generate these mPFC microcircuit adaptations, a computational model composed of 36 integrate-and-fire neurons (4 “disinhibitory” [VIP], 6 “feedback” [SST], 6 “feedforward” [PV] interneurons, and 20 pyramidal [PYR] neurons) was developed. We hypothesized that canonical patterns of microcircuit connectivity would be sufficient to generate key differences between the responses we observed in VIP, SST, PV, and pyramidal neuron activity, and that changes in the relative strength of vHPC input would explain changes in these responses over time. Therefore, neurons of each class received a combination of excitatory and inhibitory input from other classes based on characteristic connection patterns and relative synaptic weights informed by empirical studies (e.g., whole-cell electrophysiology, such as ^26,44^). All cells also received excitatory input from an external source (i.e., “vHPC”). Input from vHPC was simultaneously provided for 1 sec to all neurons in each 10-sec simulation of modeled spike output. Final model parameters were selected such that the output was qualitatively comparable to the photometry data obtained during the first IO stimulation session (40 pulses at 40 Hz condition; Figure 4B). The impact of varying the strength of vHPC input onto VIP interneurons on the evoked activity of each neuron population was then assessed.

As expected, progressively reducing vHPC input onto VIP interneurons caused a reduction in evoked VIP activity (Figure 4C,D). Interestingly, doing so also caused a robust enhancement of evoked SST interneuron activity. Further consistent with the conceptual model and photometry data, evoked PV interneuron activity decreased as vHPC input onto VIP interneurons was reduced; however, unlike the photometry data, pyramidal neuron responses also decreased. Altogether, modeling confirmed that well-established motifs of microcircuit connectivity could explain the qualitative pattern of cell type-specific responses we observed. Furthermore, selectively reducing vHPC input onto VIP interneurons may plausibly yield response changes comparable to those observed *in vivo*, at least for interneuron populations.

### Effects of reshaping vHPC input-mPFC interneuron connectivity on SWM behavior

**Figure 5.**
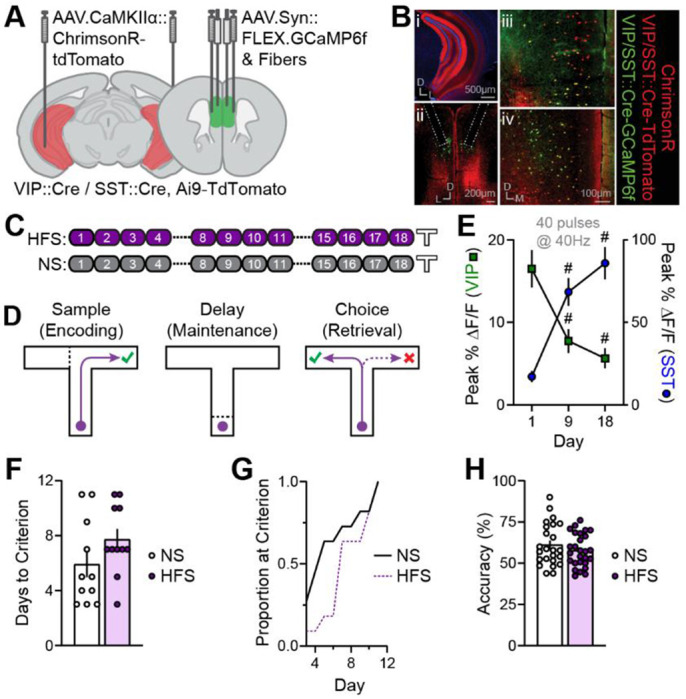
Prior vHPC input stimulation on SWM task learning. (**A**) Schematic showing AAV.CaMKIIα::ChrimsonR-tdTomato in bilateral vHPC, AAV.Syn::FLEX.GCaMP6f in bilateral mPFC, and optic fibers in bilateral mPFC of VIP or SST::Cre, Ai9-tdTomato mice. (**B**) Representative viral ChrimsonR-tdTomato expression (red) in vHPC (i) and vHPC terminals in mPFC (ii-iv); genetic tdTomato expression in VIP or SST interneurons (red cell bodies) and viral GCaMP6f expression in VIP or SST interneurons (green; ii-iv). Optic fiber placement denoted by dashed lines. DAPI staining (blue) shown in vHPC (i). (**C**) Schematic of 18-day stimulation protocol consisting of either 12 HFS sessions or no stimulation (NS) followed by training on SWM task. (**D**) Schematic of DNMS T-maze task. (**E**) Average peak stimulation-evoked Ca^2+^ responses of VIP and SST interneurons in HFS mice on Days 1, 9, and 18. Mixed-effects model, VIP Main effect of Day: F(1.237, 14.23)=20.20, p<0.0005; #p<0.005, different from Day 1; n=13. SST Main effect of Day: F(1.934, 26.11)=34.13, p<0.0001; #p<0.0001, different from Day 1; n=15. (**F**) Days to reach criterion performance (3 consecutive days ≥70% accuracy) in the SWM task in NS and HFS mice (n=11,11). (**G**) Cumulative distributions of NS and HFS mice that reached criterion across training days. (**H**) Average accuracy across training in NS and HFS mice (n=24-27).

We hypothesized that by weakening the functional connection between vHPC inputs and VIP interneurons, repeated vHPC input stimulation would impair SWM task performance and reduce task-relevant VIP interneuron activity. We further predicted that the effects on interneuron activity might be specific to particular portions of the SWM task, given that the activity and contributions of mPFC inputs and interneurons can vary within trials and across training phases ^6,29,30,34,41^. To test these hypotheses, cohorts of mice expressing ChrimsonR in vHPC and GCaMP6f in either VIP or SST mPFC interneurons received 12 HFS sessions or NS (Figure 5A–C). Similar to the findings described above (e.g., Figure 1F,I), repeated HFS without any accompanying IO stimulation strongly depressed and potentiated VIP and SST interneuron responses to vHPC input stimulation, respectively (Figure 5E). Following the last HFS/NS session, mice began training on a T-maze delayed non-match-to sample (DNMS) task (Figure 5C,D). Each trial of the DNMS task consisted of three epochs: a “sample” epoch during which mice encode information about the location of a reward; a “delay” epoch when mice must maintain spatial information; and a “choice” epoch when mice must retrieve the spatial information to choose the opposite location to receive a reward. Mice were trained for 10 trials per day with a delay length of 10 sec until they either reached a criterion of ≥70% accuracy for three consecutive days or completed 15 training days (Figure 5D).

HFS mice acquired the task at the same overall rate as their NS counterparts, despite a non-significant increase and rightward shift in the distribution of days taken to reach the performance criterion (Figure 5F,G). Prior HFS also had no effect on overall training accuracy (Figure 5H). While the high degree of variability in training performance in both NS and HFS mice may have obscured a behavioral effect of HFS, activity-induced reshaping of functional connectivity between vHPC inputs and mPFC interneurons did not consistently impair SWM task learning.

### VIP and SST interneurons show periodically divergent SWM task-related activity dynamics

**Figure 6.**
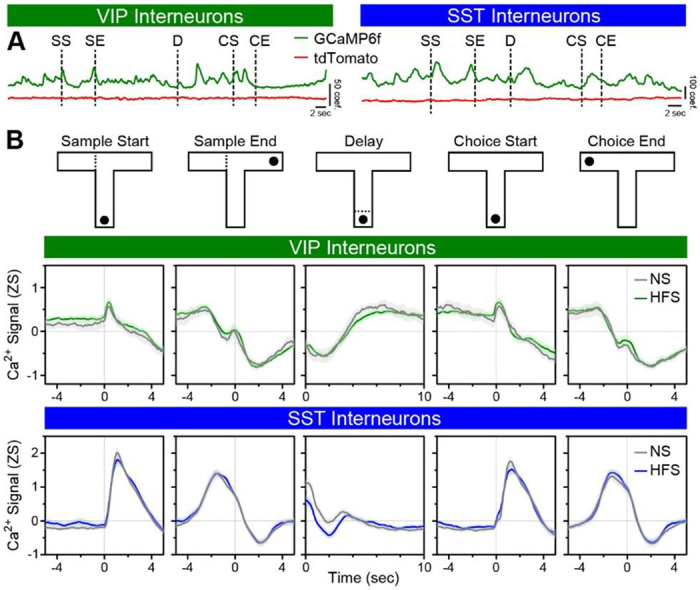
Prior vHPC input stimulation on overall SWM task-related mPFC interneuron activity patterns. (**A**) Representative VIP and SST interneurons GCaMP6f and tdTomato traces (after spectral unmixing) across a single trial. Task epochs denoted by dashed lines: Sample Start (SS); Sample End (SE); Delay (D); Choice Start (CS); Choice End (CE). (**B**) Average Z-scored Ca^2+^ signals of VIP and SST interneurons from NS and HFS mice from correct trials across all training days and aligned to discrete SWM task epochs. n=10-13 VIP; n=14-15 SST.

Next, SWM task-relevant activity profiles of VIP and SST interneurons were assessed. Using photometry data from all training days and mice, Ca^2+^ activity was derived from a validated spectral unmixing procedure ^45–47^ (Figure S5) and centered around five task events: [1] Sample Start, when mice initiated their exploration of sample arm; [2] Sample End, when mice reached the sample goal; [3] Delay, a 10-sec period initiated once mice returned to the start box; [4] Choice Start, when mice initiated movement towards the two available arms; and [5] Choice End, when mice reached the correct/incorrect choice goal (Figure 6; Figure S5E,F). VIP interneuron activity briefly increased at the beginning of the sample and choice epochs, decreased prior to and upon reaching the sample and choice goals, and increased across the delay epoch. SST interneuron activity increased as mice started the sample and choice epochs, decreased prior to and upon reaching the sample and choice goals, and decreased over the delay epoch. Together, VIP and SST interneurons showed periods of aligned and opposed activity dynamics over the course of a trial. Overall VIP and SST interneuron activity profiles differentiated between correct and incorrect trials largely and most notably after the choice goal was reached; both showed periods of reduced activity in correct trials that corresponded to reward consumption (Figure S5E). Interestingly, the two interneuron types showed dissociable relationships with velocity (Figure S5F). Cross-correlation of VIP interneuron activity with velocity revealed that these measures were negatively correlated and changes in VIP interneuron activity led changes in velocity, such that decreases in VIP interneuron activity correlated with subsequent increases in velocity (Figure S5G). In contrast, SST interneuron activity positively correlated with velocity with no discernable lead/lag relationship (Figure S5G).

### Prior vHPC input stimulation potentiates delay epoch mPFC VIP interneuron activity in early training

**Figure 7.**
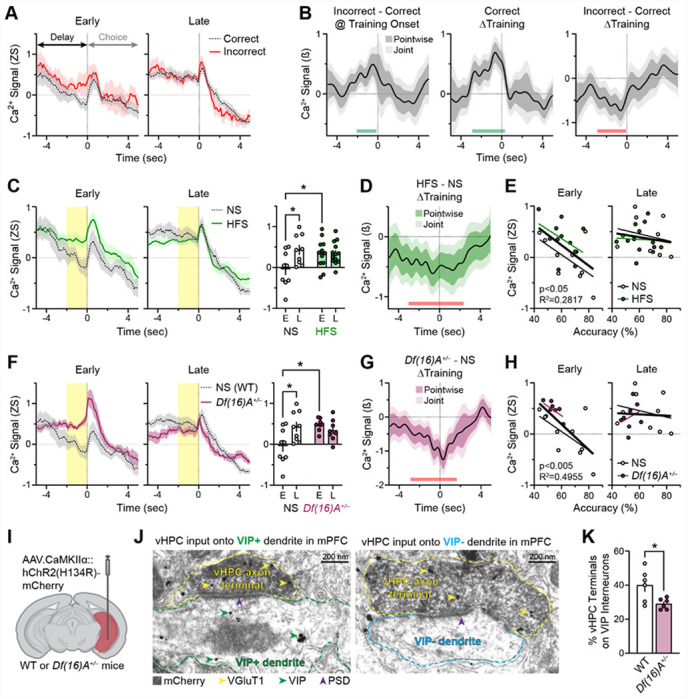
Prior vHPC input stimulation and 22q11.2 deletion syndrome-related mutation potentiate delay epoch mPFC VIP interneuron activity in early training that correlates with less effective SWM task learning. (**A**) Average Z-scored Ca^2+^ signals of VIP interneurons during the Delay-to-Choice transition for correct and incorrect trials in NS mice in early (first third of trials) and late (final third of trials) stages of training (n=7-9). (**B**) Functional linear mixed modeling (FLMM) of Outcome and Training covariate effects on trial-level VIP interneuron Ca^2+^ signals in NS mice during the Delay-to-Choice transition. Left: Functional coefficient estimates of the Outcome covariate at each timepoint in incorrect trials, showing differences in average Ca^2+^ signals in incorrect relative to correct trials at the onset of training. Middle: Functional coefficient estimates of the Training covariate in correct trials, showing changes in average Ca^2+^ signals across training. Right: Functional coefficient estimates of the interaction between the Training and Outcome covariates, showing how changes in average Ca^2+^ signals across training differ in incorrect relative to correct trials (See Figure S6A). Green and red bars denote timepoints of significant (Joint CIs do not contain Y=0) enhancement and reduction, respectively. (**C**) Average Z-scored Ca^2+^ signals of VIP interneurons during the Delay-to-Choice transition for correct trials in NS and HFS mice in Early and Late stages of training (left). Average Ca^2+^ signals in the final 2 sec of the delay epoch (“distal delay”, yellow highlighted timepoints) between Early and Late training (right). Mixed-effects model, Stimulation x Stage interaction: F(1, 19)=7.135, p<0.05; *p<0.05, different from NS Early; n=9-13. (**D**) Functional coefficient estimates of the interaction between the Training and Stimulation covariates in correct trials, showing how changes in average Ca^2+^ signals across training differ in HFS relative to NS mice. Red bar denotes timepoints of significant reduction. (**E**) Correlation of average Z-scored VIP Ca^2+^ signals during the distal delay of correct trials in Early and Late training with overall training performance (% accuracy) in NS and HFS mice. Correlation for Early training, Overall: F(1, 19)=7.453, p<0.05; R^2^=0.2817. (**F**) Average Z-scored Ca^2+^ signals of VIP interneurons during the Delay-to-Choice transition for correct trials in NS and *Df(16)A*^+/−^ mice in Early and Late stages of training (left). Average Ca^2+^ signals in the distal delay (yellow highlighted timepoints) between Early and Late training (right). Two-way ANOVA, Genotype x Stage interaction: F(1, 15)=9.657, p<0.01; *p<0.01, different from NS Early; n=8-9. (**G**) Functional coefficient estimates of the interaction between the Training and Genotype covariates in correct trials, showing how differences across training in the average Ca^2+^ signals differ in *Df(16)A*^+/−^ relative to WT mice. Red bars denote timepoints of significant reduction. (**H**) Correlation of average Z-scored VIP Ca^2+^ signals during the distal delay of correct trials in Early and Late training with overall training performance (% accuracy) in NS and *Df(16)A*^+/−^ mice. Correlation for Early training, Overall: F(1, 15)=14.73, p<0.005; R^2^=0.4955. (**I**) Schematic showing AAV.CaMKIIα::hChR2(H134R)-mCherry in unilateral vHPC of wildtype (WT) or *Df(16)A*^+/−^ mice. (**J**) Representative images of vHPC axon terminals (yellow outlines) co-expressing mCherry (dark diffused material) and VGluT1 (gold particles, yellow arrowheads) establishing asymmetric synapses (purple arrowheads denoting postsynaptic density [PSD]) on mPFC VIP+ (left, green outline; green arrowheads on VIP detected by gold particules) or VIP− (right, blue outline) dendrites. (**K**) Average percent of vHPC terminals on VIP interneurons in WT and *Df(16)A*^+/−^ mice (* unpaired t-test: t(10)=2.71, p<0.05; n=6,6).

When collectively analyzing data from all training days, mice with and without prior HFS showed statistically indistinguishable overall task-related VIP or SST interneuron activity patterns around the various SWM task events, including those spanning the sample epoch (Figure 6B). Given that the effects of HFS on task-related interneuron activity may have diminished with time (e.g., Figure 1F) or been masked by training-induced neuroadaptations, analysis was focused on activity during early-stage trials of the SWM task (first third of trials). Curiously, during correct trials in early training, VIP interneurons in NS mice showed a pronounced dip in activity during the last few seconds of the delay (“distal” delay; Figure 7A,B). This dip was smaller in early incorrect trials and absent in trials of either outcome in late training (final third of trials). Confirming these findings, functional linear mixed modeling ^48,49^ (FLMM) analysis demonstrated that distal delay VIP interneuron activity was higher in incorrect relative to correct trials at training onset, increased across training in correct trials, and increased less in incorrect trials (Figure 7B; S6A). Taken together, these data demonstrate that VIP interneuron activity during the distal delay epoch signals whether the mouse’s subsequent choice will be correct or incorrect, but only early in training.

The impact of prior vHPC input stimulation on distal delay VIP interneuron activity in early training was examined next. In early incorrect trials, prior HFS had no effect on distal delay VIP interneuron activity (Figure S6B). However, in early correct trials, prior HFS enhanced distal delay VIP interneuron activity to levels seen in incorrect trials; indeed, the dip in delay VIP interneuron activity seen in early correct trials in NS mice was absent in HFS mice (Figure 7C). Supporting these results, FLMM analysis showed that the enhancement of distal delay VIP interneuron activity across training in correct trials was blunted in HFS relative to NS mice (Figure 7D; S7). Interestingly, the magnitude of distal delay VIP interneuron activity during correct trials in early training negatively correlated with accuracy across training, such that mice with higher early-stage distal delay VIP interneuron activity showed poorer training performance; in contrast, the magnitude of distal delay VIP interneuron activity in late training had no significant relationship with accuracy during SWM task training (Figure 7E).

### Altered delay epoch VIP interneuron activity dynamics are recapitulated in a 22q11.2 deletion syndrome mouse model with SWM learning deficits

These data suggest that the dip in delay VIP interneuron activity during early correct trials is related to successful task acquisition. If so, mouse models with SWM learning deficits might lack this dip and instead show heightened VIP interneuron activity in the distal delay. To examine this, identical recordings were conducted in the *Df(16)A*^+/−^ mouse model of the 22q11.2 deletion syndrome; importantly, these mice have known structural and functional connectivity impairments in vHPC-mPFC circuits and pronounced SWM task learning deficits ^16,18,22^. Paralleling their wildtype counterparts (NS mice), *Df(16)A*^+/−^ mice expressing ChrimsonR in vHPC and GCaMP6f in mPFC VIP interneurons were trained on the SWM task without any prior stimulation. Consistent with prior reports, *Df(16)A*^+/−^ mice showed impaired training performance (Figure S6C). Recapitulating the phenotype of HFS mice, *Df(16)A*^+/−^ mice showed heightened distal delay VIP interneuron Ca^2+^ activity in early correct trials relative to NS mice; this activity remained elevated through late training (Figure 7F). Accordingly, *Df(16)A*^+/−^ mice showed blunted training-related increases in distal delay VIP interneuron activity in correct trials relative to NS mice (Figure 7G, Figure S6D). As observed in wildtype NS and HFS mice, the magnitude of distal delay VIP interneuron Ca^2+^ activity in early, but not late, correct trials in NS wildtype and *Df(16)A*^+/−^ mice negatively correlated with accuracy across training, such that mice with higher early-stage delay VIP interneuron activity showed poorer training performance (Figure 7H).

Finally, *Df(16)A*^+/−^ mice, like HFS mice, may show synaptic alterations between vHPC inputs and VIP interneurons that accompany the observed heightened delay activity. To assess such alterations, electron microscopy was used to conduct an ultrastructural analysis of the postsynaptic targeting of monosynaptic vHPC inputs in wildtype and *Df(16)A*^+/−^ mice. Mice expressing mCherry-tagged channelrhodopsin in unilateral vHPC (Figure 7I) were sacrificed and slices of their mPFC tissue were immunolabeled for three markers: mCherry (using 3,3’-diaminobenzidine) and VGluT1 (using gold nanoparticles) to label excitatory terminals of vHPC inputs, and VIP (using gold nanoparticles) to label dendrites of VIP interneurons. In serial ultrathin sections, vHPC terminals were identified and their postsynaptic dendrite was designated as VIP-positive or VIP-negative (Figure 7J). The proportion of synapses formed by vHPC inputs onto VIP-positive relative to VIP-negative dendrites was reduced in *Df(16)A*^+/−^ mice relative to wildtype mice (Figure 7K). These results suggest a reduction in synaptic targeting by vHPC inputs onto VIP neurons in *Df(16)A*^+/−^ mice.

## DISCUSSION

Here we characterized functional interactions between vHPC inputs and mPFC interneurons and uncovered synaptic and population-level plasticity between these elements. We then leveraged this plasticity—and the *Df(16)A*^+/−^ mouse model of impaired SWM learning—to inform how vHPC inputs and mPFC interneurons may cooperate to support SWM. We observed that repeated vHPC input stimulation suppressed evoked and spontaneous activity of VIP interneurons and potentiated activity of SST interneurons. *Ex vivo* whole-cell electrophysiology and computational modeling indicated that these plastic changes plausibly stem from a blunting of monosynaptic vHPC excitatory input onto VIP interneurons. In mice training on a SWM task, prior input stimulation enhanced delay VIP interneuron activity, counteracting a dip in activity normally present during correct trials early in training. Interestingly, this heightened delay VIP activity in early training correlated with poorer training performance. Finally, this activity profile was mirrored in *Df(16)A*^+/−^ mice, whose mPFC VIP interneurons we show to have reduced synaptic targeting by vHPC inputs. These findings reveal cell-type-specific plasticity at vHPC inputs onto mPFC interneurons and inform roles for VIP interneuron inputs and activity in SWM and its disease-relevant dysfunction.

### Diverse and divergent reshaping of input-interneuron interactions

Repeated stimulation of vHPC inputs to mPFC produced divergent responses in different postsynaptic populations. Specifically, VIP and PV interneuron responses were depressed, SST interneuron responses were enhanced, and putative pyramidal neuron responses remained largely stable. Informed by the *ex vivo* electrophysiology findings that repeated vHPC input stimulation can blunt vHPC monosynaptic input to VIP interneurons, we hypothesized that the various *in vivo* response adaptations may stem from this primary synaptic adaptation. Our computational model largely supported this hypothesis, showing that SST responses potentiate and PV responses depress with diminishing vHPC input to VIP interneurons. Consistent with canonical views of cortical microcircuit connectivity and other reports of *in vivo* interneuron recruitment by long-range inputs ^31,50^, our findings indicate that reduced drive of VIP interneurons can yield a net disinhibition of SST interneurons that in turn silences PV interneurons. The synaptic alterations underlying the potentiation of SST interneuron responses to minimal IO stimulation (Figure 1H–I; S3E–H) are unclear and may differ from those induced by the more intensive vHPC input HFS. However, this potentiation of SST interneuron responses did parallel modest depression of VIP interneuron responses to the same minimal stimulation (Figure S1E–F; S3A–D), suggesting that subtle changes in VIP interneuron recruitment by long-range inputs may yield pronounced changes in SST interneuron activity.

### vHPC inputs may not be primary drivers of SWM task-related VIP or SST interneuron activity

Despite the dramatic stimulation-induced reshaping of functional connections between vHPC inputs and mPFC interneurons (e.g., ~65% decrease in VIP interneuron responses and ~500% increase in SST interneuron responses; Figure 1E–J; Figure 5E) and altered spontaneous VIP and SST interneuron activity in non-behaving mice (Figure 2), task-related VIP and SST interneuron activity patterns were largely unaltered during SWM performance. The stability of the activity profiles during the sample epoch was particularly striking, given that the activity of vHPC inputs and SST interneurons are independently required to support the encoding of task-relevant spatial information in the mPFC ^6,41^. These findings suggest that vHPC inputs are not primary drivers of SWM task-related VIP or SST activity, and that other synaptic and circuit mechanisms predominated in generating the observed overall interneuron activity profiles. While these results reflect a stability in the averaged population activity, alterations in the activity of individual or ensembles of interneurons could occur following repeated vHPC input stimulation ^29^. Moreover, the stability of these activity profiles, similar to the stability of the average pyramidal neuron responses to vHPC input stimulation, may reflect slowly developing, compensatory adaptations in highly interconnected mPFC microcircuits that serve to constrain population-level neural dynamics that support SWM^52^. Indeed, evidence for the contributions of vHPC inputs and mPFC interneurons to SWM and other delayed-response tasks derives from acute, temporally restricted manipulations (e.g., epoch-restricted optogenetic inhibition) that are unlikely to trigger broad counteradaptations ^6,30,35,41^. Future work should leverage novel tools ^51^ that enable acute modulation of synaptic connections between defined pre- and postsynaptic elements with comparable temporal specificity.

### “Interference by irrelevance”: how heightened delay VIP interneuron activity may impede SWM task training

In this study we characterized mPFC VIP interneuron dynamics from the onset of SWM task training, yielding novel insights into how VIP interneurons may support, or impede, SWM task training. Owing to their preferential targeting of other inhibitory neurons in cortical microcircuits, VIP interneurons play a central role in disinhibiting pyramidal neuron activity ^32^. Accordingly, VIP interneuron activity in the delay epoch of previously acquired spatial and non-spatial WM tasks has been shown to amplify pyramidal activity dynamics that encode task-relevant information and thereby improve task accuracy ^34,35^. However, early in task training, before animals have acquired the task rule, pyramidal activity dynamics encode less task-relevant information ^53–55^. Therefore, if delay VIP interneuron activity is high early in training, task-irrelevant pyramidal neuron dynamics may be amplified, leaving animals more likely to make incorrect choices. Conversely, if delay VIP interneuron activity is low early in training, task-irrelevant dynamics may be unamplified and therefore less prone to interfere with adaptive decision-making. In line with this perspective, which may be labeled “interference by irrelevance”, we found that in early task training, VIP interneuron activity was higher in the delay epochs of incorrect relative to correct trials (Figure 7A,B). In our binary delayed non-match-to-sample SWM task, lower delay VIP interneuron activity in early training may minimize interference and thus enable mice to preferentially follow their innate tendency to explore the newly available arm ^56^, yielding a correct choice.

Our results further show that manipulations that worsen SWM training performance enhance delay VIP interneuron activity in correct trials to levels seen in incorrect trials, and that this heightened VIP interneuron activity is anti-correlated with training accuracy. These findings may also be understood through the “interference by irrelevance” framework. Early in training, correct responses often arise by chance (or through innate exploratory behavior) rather than from an understanding of the task rule. Therefore, correct trials can occur even when delay pyramidal neuron activity encodes task-irrelevant information. On correct trials when delay VIP interneuron activity is high and thus pyramidal neuron activity encoding task-irrelevant information is amplified, mice may attribute their correct choice to this irrelevant information. This misattribution may cause mice to make more errors over the course of training. In this way, heightened delay VIP interneuron activity in early training may maladaptively amplify task-irrelevant pyramidal neuron activity dynamics, thus worsening training performance.

### Potential neuroadaptations underlying elevated delay VIP interneuron activity in early training

Unexpectedly, we found that prior vHPC input stimulation that weakened monosynaptic connectivity of vHPC inputs onto VIP interneurons produced an increase in VIP interneuron activity in the distal delay in early training. However, many potential parallel or secondary neuroadaptations may explain this seemingly paradoxical finding. For example, weakening vHPC inputs onto VIP interneurons may result in secondary increases in the efficacy of inputs from other excitatory inputs, such as those from the mediodorsal thalamus (MD); such increases could maladaptively amplify VIP interneuron activity (and task-irrelevant activity) in early training ^26,57–59^. Interestingly, *Df(16)A*^+/−^ mice, which displayed comparably enhanced delay VIP interneuron activity to HFS mice, also showed synaptic alterations between vHPC inputs and VIP interneurons; specifically, the proportion of synapses formed by vHPC inputs onto VIP-positive relative to VIP-negative dendrites was found to be reduced in *Df(16)A*^+/−^ mice. The convergence of functional and structural adaptations in vHPC input-stimulated mice and *Df(16)A*^+/−^ mice further reinforce the possibility that reweighting of excitatory inputs onto VIP interneurons may underlie the altered delay-related activity dynamics of these neurons. Alternatively, neuroadaptations in prefrontal dopamine signaling, long implicated in sustaining pyramidal neuron dynamics to support SWM maintenance ^60–62^ may account for the heightened delay-epoch VIP interneuron activity in early training. Indeed, abnormally heightened mPFC D1 receptor signaling in VIP interneurons, whether through adaptations downstream of repeated vHPC input activation ^63,64^ or *Df(16)A*^+/−^ mutation ^65^, could preferentially enhance VIP interneuron activity in distal portions of the delay epoch ^34,66^ and impair SWM task performance^67^. The precise mechanisms of the altered delay VIP interneuron activity remain speculative and warrant future investigation.

In conclusion, we uncovered cell-type-specific plasticity within intact vHPC-mPFC circuits and leveraged it to inform how discrete circuit elements interact to mediate cognitive function and disease-relevant dysfunction. The striking malleability we observed in input-interneuron connectivity has important implications, not only for the design and interpretation of *in vivo* optogenetics experiments, but also for translational studies seeking to harness the profound control of inhibitory microcircuits over local and long-range neural communication. Moreover, our findings underscore the determinative role behavioral state can play in the expression of such plasticity. Indeed, as we show, the same synaptic adaptation that blunts neuronal activity in one behavioral state may amplify it in another. Lastly, by comparing neurophysiological signatures of activity-induced and disease-relevant genetic neuroadaptations, our work advances novel, testable hypotheses of the circuit basis of disordered cognition, and highlights VIP interneuron inputs and activity as compelling targets for potential cognitive therapies.

## METHODS

### Mice.

C57BL/6J (000664), VIP::Cre (031628), SST::Cre (013044), PV::Cre (017320), SST::Flpo (031629), and Ai9-tdTomato (007909) mice were obtained from The Jackson Laboratory (Bar Harbor, ME). Mice homozygous for the VIP, SST, or PV::Cre transgenes were crossed with C57BL/6J mice to generate mice used in the opto-photometry experiments characterizing the effects of vHPC input stimulation on mPFC interneuron activity. Mice homozygous for the VIP::Cre transgene were crossed with mice homozygous for the SST::Flpo transgene to generate mice used in the slice electrophysiology experiments. Mice homozygous for the VIP::Cre or SST::Cre transgene were crossed with mice homozygous for the Ai9-tdTomato Cre reporter transgene to generate mice used in the SWM experiments. *Df(16)A*^+/−^ mice were obtained from the laboratory of Dr. Joseph Gogos at Columbia University. *Df(16)A*^+/−^ mice were backcrossed for over 10 generations with C57BL/6J mice prior to obtaining them from Columbia University and were maintained by *Df(16)A*^+/−^ x C57BL/6J crossings throughout the present experiments. Male *Df(16)A*^+/−^ mice were crossed with female mice homozygous for both VIP::Cre and Ai9 to yield the triple-transgenic mice (and their *Df(16)A*^+/+^ wildtype counterparts) used in the SWM experiments.

Mice were group-housed with littermates (up to 5 mice/cage) in a temperature- and humidity-controlled National Institutes of Health animal facility on a 12-h light–dark cycle. Except when food-restricted for behavioral training (see SWM Task), all mice were given *ad libitum* access to food and water. All procedures were performed in accordance with the US National Research Council Guide for the Care and Use of Laboratory Animals and were approved by the National Institute of Neurological Disorders and Stroke, National Institute of Mental Health, and National Institute on Drug Abuse Animal Care and Use Committees.

### Surgical Procedures.

#### Surgical Preparation.

Adult mice (2.5-8 months) were anesthetized with 3-5% isoflurane (v/v in oxygen, flow rate of 1 L/min) in an induction box until sedated, weighed, shaved at the surgical site with hair clippers, and placed in a stereotaxic apparatus (Kopf Instruments, Model 900) using non-rupture ear bars (Model 922). Sterile ophthalmic ointment (Paralube) was placed on mouse eyes to prevent corneal drying. The scalp was disinfected with alternating applications of Betadine and 70% isopropyl alcohol. Mice were maintained at 0.8-2% isoflurane via a nose-cone adaptor on a 45°C heating pad (TC-1000, CWE) for the duration of the surgery. Prior to and during the surgery, anesthesia depth was tested by a toe pinch by the surgeon’s fingers; mice with any response were given supplemental isoflurane. Tips of all surgical instruments were sterilized in a hot bead sterilizer (Braintree Scientific) and cooled prior to surgery.

#### Virus injection.

A midline scalp incision was made and the skull was exposed. Nose position was adjusted to level bregma and lambda (±0.05 μm in the D/V plane). For the opto-photometry experiments, burr holes were drilled above unilateral vHPC (in mm, A/P: −3.5, M/L: ±3.35) and ipsilateral mPFC (A/P: +1.9, M/L: ±0.4) of VIP, SST, or PV::Cre mice. Adeno-associated viruses AAV1.Syn::ChrimsonR-tdTomato (Addgene, 59171) or AAV1.CAG::tdTomato (Addgene, 59462), diluted in sterile saline to a titer of 5x10^12^ GC/mL, were administered to the vHPC (D/V: −3.25 from brain surface) at a volume of 700nL through a pulled (Narishige, PC-100) glass pipette (World Precision Instruments, 1B100F-4) connected via a needle adaptor (Colbert Associates Lab Store, 55750-01) to a syringe (Hamilton, Model 75RNSYR). AAV9.Syn::FLEX.GCaMP6f (Addgene, 100833), AAV9.CaMKII::GCaMP6f (Addgene, 100834), or AAV5.hSyn::DIO.EGFP (Addgene, 50457) at titers of 2.5x10^12^−2.8x10^13^ GC/mL were administered to the mPFC (D/V: −1.45) at a volume of 500 nL through a separate identical injection system. For the slice electrophysiology experiments, burr holes were drilled above bilateral vHPC (A/P: −3.5, M/L: ±3.35) and bilateral mPFC (A/P: +1.9, M/L: ±0.4) of VIP::Cre;SST::Flpo mice. AAV1.Syn::ChrimsonR-tdTomato (Addgene, 59171, 5x10^12^ GC/mL) was injected into vHPC (D/V: −3.25, 600nL). A combination AAV9.CAG::FLEX.tdTomato (Addgene, 51503) and AAV9.EF1a::fDIO.EYFP (Vector Biolabs) with final titers of 1.7x10^12^ and 1.25x10^12^ GC/mL, respectively, was injected into mPFC (D/V: −1.45, 500 nL). For the SWM experiments, burr holes were drilled above bilateral vHPC (in mm, A/P: −3.5, M/L: ±3.35) and bilateral mPFC (A/P: +1.95 , M/L: ±0.7, at a 15° angle) of VIP::Cre;Ai9-tdTomato or SST::Cre;Ai9-tdTomato mice (with or without the *Df(16)A*^+/−^ mutation). Two injections of AAV5.CaMKII::ChrimsonR-tdTomato (Vector Biolabs, 2x10^12^) per hemisphere were injected into vHPC (D/V: −4.25 and −3.75 from bregma, 1 μL at each depth) at a rate of 100 nL/min using a syringe pump (Quintessential Stererotaxic Injector, Stoelting). AAV9.Syn::FLEX.GCaMP6f (Addgene, 100833, 2.8x10^13^) was manually injected into mPFC (D/V: +1.8 from bregma, 500nL) over the course of 10 min using a Hamilton syringe and a 32-gauge needle (Model 7001KH). In all cases to facilitate viral diffusion, the pipette was kept in place for at least 5 min post-infusion before being slowly withdrawn. Mice used in the opto-photometry and slice electrophysiology experiments had their incision sealed with tissue adhesive (Vetbond, 146Sb, 3M), were returned to clean homecages, and were left to recover for 8-13 weeks prior to optic fiber implantation. Mice used in the SWM experiments underwent optic fiber implantation immediately following viral infusion.

### Fiber implantation.

For mice in the opto-photometry and slice electrophysiology experiments, mice were anesthetized, the surgical site was prepared, and a midline scalp incision was made to expose the skull, as described above. Unilateral (opto-photometry) or bilateral (slice electrophysiology) holes were drilled above mPFC (A/P: +1.9, M/L: ±0.4). Two miniature screws (Antrin Miniature Specialties, Inc) were threaded into the skull (approx. A/P: −1.0, M/L: ±2.5). After piercing dura with a 30-G needle, unilateral (opto-photometry) or bilateral (slice electrophysiology) optic fibers (200-μm core, 0.39 NA; ThorLabs) were slowly lowered into the mPFC (D/V: −1.0 from brain surface). For the SWM experiments, bilateral optic fibers (200-μm core, 0.39 NA) were implanted at a 15° angle (DV: −1.6 from bregma). Optic fibers and screws were secured to the skull with dental cement (Unifast Trad or 3M RelyX Unicem Cement Automix, Henry Schein).

### Postoperative procedures.

Ketoprofen (5-10 mg/kg) and sterile saline (1 mL) were administered 30 min prior to the end of surgery. Mice were rehoused with one or more littermates in clean cages, except for those used in the SWM experiments, which were single-housed after surgery. Ketoprofen and saline injections were administered 24- and 48-h post-surgery. Mice were left to recover for at least 10 days prior to beginning experiments.

### Photometry systems.

Custom-built spectrometer-based systems (based on published systems^45–47^) were used to conduct fiber photometry recordings. For the opto-photometry experiments, blue light from a 473-nm laser (MBL-III-473-100mW, Ready Lasers) passed through a Noise Eater (NEL01, ThorLabs), and red light from a 635-nm laser (MRL-III-635L-200mW), were directed into a kinematic fluorescence filter cube (DFM1, ThorLabs) onto a quadruple-edge dichroic mirror (ZT440/488/561/635rpc, Chroma). Light was then coupled using an FC/PC fiber coupler (PAF2-A4A, ThorLabs) into a fiber patchcord (200-μm core, 0.39 NA, ThorLabs) connected to an optic fiber rotary joint (FRJ_1x1_FC-FC, Doric Lenses) followed by another patchcord (200-μm core, 0.48 NA, Doric). Blue light power was approximately 80 μW at the ferrule end of the final patchcord, resulting in approximately 70-μW output from the surgically implanted ferrule. To pilot the optogenetic stimulation parameters, red light pulses were delivered with power approximately 1.2-8.4 mW at the end of the final patchcord, resulting in approximately 1-7-mW output from the implanted ferrule (Figure S1B); 5-mW output was used for all subsequent experiments. On each recording day, the top of the surgically implanted ferrule was cleaned with 70% ethanol and lens paper (Ted Pella) and securely attached to the ferrule end of the final patchcord via a mating sleeve (Precision Fiber Products). Fluorescence emission from brain tissue was collected by the same fiber, filtered through a triple-band emission filter (ZET488/561/633m, Chroma), and directed using a fiber coupler (PAF2S-11A, Thorlabs) into a 200-μm core, anti-reflection-coated fiber (M200L02S-A, ThorLabs) which led to a spectrometer (QEPro, Ocean Insight). The spectrometer quantified photon counts across a 350–1130 nm wavelength window when triggered by an external TTL. A Python-controlled waveform generator (PulsePal v2, SanWorks) delivered 40-Hz TTLs to the spectrometer to trigger 17-ms spectral integration events and 1-40-Hz TTLs to the red laser to trigger 5-ms light pulses. The spectrometer TTLs were delayed by 5ms relative to the red laser TTLs to ensure that red light pulses ended prior to spectral integration events and thus did not contaminate the recorded spectra. The waveform generator also delivered 20-Hz TTLs to the camera (FLIR Blackfly S USB3) to trigger the shutter. Video frames generated at 20 Hz were processed using Bonsai software operating real-time DeepLabCut processing nodes ^68,69^ such that environmental and mouse features were assigned coordinates for each video frame as it was captured. Camera shutter events (also 20 Hz) were simultaneously captured as digital events in OpenEphys to facilitate subsequent alignment of photometry and positional data.

The SWM experiments used a comparable spectrometer-based photometry system with a few modifications. Blue light only was directed onto a dual-edge dichroic mirror (ZT488/561rpc, Chroma) and fluorescence emission from brain tissue was filtered through a dual-band emission filter (ZET488/561m, Chroma). The waveform generator delivered 20-Hz TTLs to the spectrometer to trigger 37-ms spectral integration events. 20-Hz TTLs were simultaneously delivered to the camera to trigger the camera shutter at the precise moments of spectral integration.

### Optogenetic stimulation parameters.

Over 50 days, mice used in the opto-photometry experiments received six “input-output” (IO) sessions (Days 1, 8, 15, 22, 29, and 50) to characterize response curves of each mPFC neuron population to vHPC input stimulation. Each IO day began with 10 min of baseline photometry recordings (used to assess changes in spontaneous Ca^2+^ activity). Following this baseline period, vHPC terminals were stimulated with 5-ms pulses of red light across a range of pulse numbers and frequencies. Specifically, mice received two rounds per IO day of the following stimulation conditions: 5 Hz (1, 5, and 10 pulses); 10 Hz (1, 5, 10, and 20 pulses); 20 Hz (1, 5, 10, 20, and 30 pulses); and 40 Hz (1, 5, 10, 20, 30, and 40 pulses). Each stimulation condition was delivered as a 30-sec “trial” including a 10-sec pre-stimulation period and 19-sec post-stimulation period. For 12 days early in the 50-day timeline (Days 4-7, 11-14, and 18-21), a subset of mice received additional “high-frequency stimulation” (HFS) of vHPC inputs intended to induce plasticity in the response curves characterized on IO days. Specifically, “HFS” mice received 100 trains of 40 5-ms pulses delivered at 40-Hz (delivered 30 sec apart) on each of the 12 HFS days. “No HFS” mice received all the same conditions (e.g., handling and tethering, time in cylindrical chamber, blue light to enable photometry recordings), but did not receive red light pulses. In all experiments involving optogenetic stimulation, red laser pulses were 5 ms long and laser power was calibrated to be ~5mW at the tip of the implanted ferrule.

A separate cohort of mice was used to confirm whether IO stimulation alone was sufficient to induce changes in SST interneuron responses (Figure S3). A subset of these mice received IO stimulation paired with photometry on days 1, 8, 15 and 22 (“D1-22”). Other mice underwent photometry recordings (and thus identical handling, time in chamber, and blue light) on each of these days but received IO stimulation only on Day 22 (“D22 Only”).

Mice used in the slice electrophysiology experiments received either 100 trains of 40 pulses at 40 Hz for 12 days (Days 1-4, 8-11, and 15-18; HFS mice), or identical handling, tethering, and chamber exposure but without red light pulses (No Stimulation [NS] mice). Similarly, mice in the SWM experiments received 12 days of 100 trains of 40 pulses at 40 Hz (HFS mice), or identical conditions without red light pulses (NS mice). To enable characterization of HFS-induced changes in stimulation-evoked interneuron responses in mice used in the SWM experiments, on Days 1, 9 and 18, HFS mice received 50 trains of unilateral stimulation during simultaneous photometry recordings (conducted sequentially for both hemispheres), followed immediately by 50 trains of bilateral stimulation without photometry recording. NS mice received identical conditions but without red laser pulses. The hemisphere that showed the most robust and characteristic GCaMP + tdTomato spectral features and the most pronounced changes in evoked Ca^2+^ response across HFS days (depression for VIP interneurons, potentiation for SST interneurons) was selected for photometry recordings during subsequent SWM task training.

The results of several early pilot studies justified the use of the above stimulation paradigms that focus on assessing between-day, rather than within-day, changes in stimulation-evoked responses. In the pilot studies, each day for 8-10 days, SST::Cre mice with ChrimsonR in vHPC and Cre-dependent GCaMP6f in mPFC received two rounds of IO stimulation before and two rounds after 80 trials of HFS (40 pulses at 40 Hz, trials separated by 30 sec). Pre-HFS evoked responses largely matched post-HFS evoked responses within a given day; however, both pre- and post-HFS evoked responses potentiated across days (e.g., from Day 1 to 9).

### Optogenetic stimulation without photometry recording.

Bilateral red laser illumination without simultaneous photometric recordings was conducted on select days for the slice electrophysiology and SWM experiments. Red light from a laser (MRL-III-635L-200mW, Ready Lasers) was directed through a fiber coupler (PAF2S-18A, ThorLabs) into a fiber patchcord (200-μm core, 0.39 NA, ThorLabs) connected to an optic fiber rotary joint (FRJ_1x2_FC-FC, Doric Lenses) followed by dual patchcords (200-μm core, 0.48 NA, Doric) connected to surgically implanted ferrules. Illumination was delivered in cylindrical containers identical to those used in the opto-photometry experiments.

### Slice electrophysiology.

#### Acute brain slice preparation.

Mice were anesthetized using Euthanasia solution (sodium pentobarbital and phenytoin; NIH Veterinarian Services) and decapitated. Brains were quickly removed and placed in ice-cold cutting solution containing (in mM): 92 NMDG, 20 HEPES, 25 glucose, 30 NaHCO_3_, 2.5 KCl, 1.2 NaPO_4_ saturated, 10 Mg-sulfate, and 0.5 CaCl_2_ with 95% O_2_/5% CO_2_ (osmolarity 303–306 mOsm, Wescorp). The extracted brain was promptly blocked, dried on filter paper, and affixed to a platform immersed in ice-cold NMDG-based cutting solution within a vibratome chamber (Leica VT1200). Coronal slices (250-μm thick) containing mPFC were cut at 0.07 mm/s. Slices were incubated in a chamber containing a NMDG-based cutting solution for 5-10 min at 34°C, and then transferred to a chamber filled with a modified holding aCSF saturated with 95% O_2_/5% CO_2_. The solution contained (in mM): 92 NaCl, 20 HEPES, 25 glucose, 30 NaHCO_3_, 2.5 KCl, 1.2 NaPO_4_, 1 mM Mg-sulfate, and 2 mM CaCl_2_ with osmolarity of 303–306 mOsm, at room temperature for a minimum of 1 h. Slices were kept in the holding solution until being transferred to the recording chamber.

#### *Ex vivo* whole cell electrophysiology.

Whole-cell patch-clamp electrophysiology studies were conducted following previously described methodology ^70–72^. Cells were visualized using infrared differential interference contrast (IR-DIC) optics on an inverted microscope (Olympus BX5iWI). The recording chamber was perfused at a rate of 1.5–2.0 ml/min with artificial cerebrospinal fluid (aCSF) consisting of (in mM): 126 NaCl, 2.5 KCl, 1.4 NaH_2_PO_4_, 1.2 MgCl_2_, 2.4 CaCl_2_, 25 NaHCO_3_, and 11 glucose (303-305 mOsm), using a perfusion pump (World Precision Instruments). For whole-cell recordings of AMPA and NMDA currents, glass microelectrodes (3–5 MΩ) were used, containing (in mM): 117 cesium methanesulfonate, 20 HEPES, 0.4 EGTA, 2.8 NaCl, 5 TEA-Cl, 4 Mg-ATP, and 0.4 Na-GTP (280-285 mOsm). SST-positive and VIP-positive cells were identified based on the presence of tdTomato and GFP fluorescence, respectively. Neurons were voltage-clamped using a Multiclamp 700B amplifier (Molecular Devices). Data were filtered at 2 kHz and digitized at 20 kHz with a 1440A Digidata Digitizer (Molecular Devices). Series resistance (<20 MΩ) was monitored using a −5 mV voltage step; cells with >20% change in series resistance were excluded from further analysis. Monosynaptic oEPSCs evoked by vHPC input stimulation (1-ms pulses, 10-sec inter-pulse interval) were isolated using TTX (1 μM, Tocris) and 4-AP (50 μM, Tocris). For biophysically isolated AMPA and NMDA currents, cells were held at −70 mV and +40 mV, respectively. NMDA current peaks were detected between 60 ms after the onset of red-light stimulation. For pharmacologically isolated AMPA and NMDA currents, cells were held at +40 mV, and NMDA currents were digitally subtracted from recording with the presence of D-AP5 (50 μM, Abcam).

### Spatial working memory assay.

#### Maze apparatus.

A custom-built automated T-maze was used for assessing SWM task learning. Each arm of the T-maze was 12.7 cm wide and 30.5 cm high. The center arm (stem) was 60 cm long (including an 18-cm start box), and the goal arms were 26.7 cm long. The start box and goal arms contained a reward port for delivery of sweetened condensed milk (~50 μl, 20% in deionized water) through a blunt 25G needle. Delivery was triggered when mice broke infrared beams positioned in the maze walls during task-relevant periods. A circular rotary platform, positioned such that one half comprised the choice point of the maze, made 180° rotations during the delay epochs of each trial to prevent mice from using scent cues to guide their subsequent choice (described below). Maze components were controlled using custom Python scripts.

#### SWM task.

Single-housed mice post-surgery were placed on a food-restricted diet consisting of 1.5-3g of mouse chow daily to maintain their weight at 80%-85% pre-restriction weight (after correcting for weight of implanted optic fibers). One day following completion of the stimulation protocol, mice were food restricted for three days before they underwent two daily sessions of maze habituation in which they freely visited all arms (baited with milk solution) in the T-maze for 30 min. Mice were tethered to the optical patch cord on the second habituation session (and all sessions thereafter). Mice underwent two consecutive daily sessions of shaping trials during which mice visited a single available arm (randomized left/right) for milk reward, returned to the start box for milk reward. Mice were contained in the start box for 10 sec before visiting the alternate single available arm (forced choice) for milk reward. Shaping trials had a 20-sec inter-trial interval (ITI) and shaping sessions consisted of up to 10 trials over 30 min. To advance to the training phase, mice had to complete 10 shaping trials in 30 min on two days. Mice then began training on a delayed non-match-to-sample task (DNMS) of SWM. Each DNMS trial consisted of 3 epochs: Sample, Delay, and Choice. In the sample epoch, mice visited a single available arm (randomized between left/right) for milk reward and returned to the start box. Mice were contained in the start box for 10-sec delay period, after which both goal arms became available and mice had to choose to visit the goal arm not visited during the sample epoch to obtain milk reward. Trials were separated by an ITI of 20 sec. Mice performed 10 trials per daily training session until reaching a criterion of three consecutive days of ≥70% correct trials or completing 15 training days. All SWM task training was performed during the light cycle. Experimenters conducting behavior training and testing were blinded to mouse stimulation condition and *Df(16)A*^+/−^ mutation.

#### Photometry during optogenetic stimulation and SWM task learning.

Photometry and position data were recorded throughout optogenetic stimulation sessions and SWM training as described above. Briefly, synchronized 40- or 20-Hz pulses delivered to the spectrometer and camera were coupled with Bonsai-mediated real-time DeepLabCut annotation of environmental features and mouse position alongside OpenEphys-mediated capture of corresponding 40- or 20-Hz digital timestamps. Stimulation events (e.g., start/stop of stimulation trials, trial-specific stimulation parameters) and maze events (e.g., choice start) were simultaneously captured as text network events in OpenEphys and subsequently integrated with photometry and position data. Photometry and position data streams were paused for 2 sec between each 30-sec stimulation trial (bridging the ~20 sec post-stimulation recording with the 10-sec pre-stimulation recording of the next stimulation trial) or each SWM task trial (spanning the 8-10 sec timepoint of each 20-sec ITI). These 2-sec gaps enabled brief openings of a Python-controlled, servo-driven clamp on the optic fiber rotary joint (www.thingiverse.com/thing:2661755). The clamp prevented light artifacts due to rotary joint movement during recordings. The brief openings allowed for any accumulated patchcord tension to be released prior to reclamping and resumed recording.

#### Behavioral data processing.

Bonsai-DLC was used for behavioral tracking ^68,69^. Bonsai-DLC enabled use of pre-trained DLC models in a Bonsai workflow to process live-streamed videoframes and generate DLC coordinates in real time. DLC models were created by labeling 300–500 frames of videos containing the cylindrical chamber or T-maze, comprised of approximately 10–20 frames from each of 20–30 videos of different mice with comparable optic fiber implants/patchcords to experimental mice. In addition to cylinder/maze boundaries and landmarks, six mouse body parts were labeled on each frame: nose, headcap, shoulder, midpoint, hind, and base of the tail. The midpoint label was used for behavioral analysis. The model was trained for approximately 750,000–1,000,000 iterations, yielding confidence values of >99% in most cases. A Python-controlled waveform generator (PulsePal v2, SanWorks) delivered 20-Hz TTLs to an FLIR Blackfly S USB3 camera. Each resulting frame was processed for all model-labelled chamber/maze/body parts. A confidence threshold of ≥95% was applied in Bonsai to the positional data. Exported data were down-sampled to 10 Hz, and a Kalman Filter (pykalman.github.io) was applied to estimate XY position at all timepoints, including where position values were missing (i.e., those with confidence <95%). XY position was converted from pixels to cm and used to calculate velocity (cm/sec).

#### Photometry data processing.

The spectrometer-based photometry systems recorded a fluorescence spectrum for each timepoint (e.g., 20 or 40 Hz, depending on the TTL trigger rate of the experiment). In all cases, the shape and amplitude of the fluorescence spectra were used to confirm *in vivo* GCaMP6f and tdTomato expression in each mouse on each day. The GCaMP and tdTomato portions of each spectrum were defined as spanning 500-541 nm and 577-618 nm, respectively. Photon counts within these wavelength ranges were summed to generate GCaMP and tdTomato photon count timeseries.

#### Opto-photometry photometry processing.

In the opto-photometry experiments, the GCaMP6f and tdTomato photon count timeseries were linear regression-corrected to remove gradual reductions in signal due to fluorophore signal fading across each recording session. The timeseries were downsampled from 40 to 10 Hz. %ΔF/F of the GCaMP signal was calculated for each stimulation “trial” using the average photon counts in the 10-sec pre-stimulation period. %ΔF/F values were averaged across trials with identical stimulation parameters (e.g., two trials of each stimulation condition on IO days; 100 trials of the sole condition on HFS days), except for analyses to assess within-session changes in response magnitude. The peak GCaMP %ΔF/F value of each processed signal within the 10-15-sec window was taken as the stimulation-evoked Ca^2+^ response magnitude. For assessing stimulation-induced changes in endogenous Ca^2+^ activity, recordings during the four 2.5-min bins of the 10-min baseline periods that preceded each IO stimulation day were used. %ΔF/F was calculated for each of the 2.5-min bins using their respective average GCaMP photon counts. Python’s “find_peaks” function was implemented to identify (for each bin, subsequently averaged) the magnitude, frequency, and half-width of significant Ca^2+^ events (threshold: 2.91 x median absolute deviation [approximating the 95% confidence interval for Gaussian data]; min width: 0.5 sec; max width 10 sec; min interval between peaks: 1 sec; window length peak features: 1.6 sec). These values were normalized to those of IO Day 1.

As expected, the tdTomato signal in mice used in the opto-photometry experiments was typically low since it constituted that of only the diffuse ChrimsonR-expressing vHPC inputs to the mPFC. This precluded spectral unmixing of the GCaMP and tdTomato signals in the opto-photometry experiments. That said, motion-related artifacts did not confound these experiments. Indeed, not only was mouse movement within the cylindrical chamber minimal (averaging ~1.3 cm/s during IO sessions), but motion-related artifacts of any significance were ruled out by extensive piloting and photometry recordings of mice expressing control fluorophores showing highly stable (i.e., within range of −2 to +2 %ΔF/F) signals as mice moved around the cylindrical chamber (EGFP, Figure S1D,E; tdTomato, data not shown).

#### Spectral unmixing for SWM experiments.

In the SWM experiments, VIP::Cre;Ai9-tdTomato and SST::Cre;Ai9-tdTomato mice were intentionally used to ensure robust GCaMP and tdTomato signals, such that spectral unmixing of the two could be performed. To separate the fluorescence derived from GCaMP6f and tdTomato, all raw emission spectra were transformed using a spectral linear unmixing algorithm written in R, as described previously ^45–47^. The resulting unmixed GCaMP6f and tdTomato coefficient timeseries (example traces in Figure S5D) were linear regression-corrected to remove gradual reductions in signal due to fluorophore signal fading across the behavioral test. To control for potential movement artifacts in the fluorescence signal, the ratio of the unmixed, linear regression-corrected GCaMP6f and tdTomato coefficients was calculated ^45–47^. The GCaMP:tdTomato ratio timeseries were then down sampled from 20Hz to 10Hz, aligned to positional data from DeepLabCut and maze event data from OpenEphys, and split into individual trials. Z-scores of this ratio were then calculated for each 10-Hz time point using the mean and standard deviation of its corresponding full trial. These z-scores, referred to as “Ca^2+^ Signal (ZS)”, were then aligned to (and averaged across) peri-event periods (e.g., sample start, sample end, delay, choice start, choice end). Z-scores of VIP interneuron Ca^2+^ signals were averaged in the 2-sec window prior to the “Choice Start” event to assess group differences in “distal” delay epoch-related activity. For assessing how these signals varied across training, averaged values from the first third of trials were deemed “early” and the final third of trials were deemed “late” for each mouse.

#### Additional processing.

To facilitate visual comparison of task-relevant VIP and SST interneuron Ca^2+^ signals and their association with mouse velocity (Figure S5E), Ca^2+^ signals (z-scored) and velocities (cm/sec) were normalized such that data from individual trial events (e.g., single “Sample Start” events) ranged from +1 to −1. These normalized Ca^2+^ and velocity data were then averaged within a given mouse. Cross-correlations of VIP and SST interneuron Ca^2+^ signals with their respective simultaneous velocity measurements were conducted on data from full individual trials with a maximum lead/lag value of 50 (±5 sec of 10 Hz timeseries; Figure S5F).

#### Electron Microscopy.

We used cohorts of mice (6 *Df(16)A*^+/−^ and 6 wildtype mice) injected with AAV5-CaMKIIa-hChR2(H134R)-mCherry into the right vHPC for detection of mCherry and VGluT1 in axon terminals in mPFC by electron microscopy. Vibratome tissue sections (40 μm) were rinsed with phosphate-buffered saline (PBS), incubated with 1% sodium borohydride in PBS for 30 min to inactivate free aldehyde groups, rinsed in PBS, and incubated with blocking solution (1% normal goat serum [NGS], 4% BSA in PBS supplemented with 0.02% saponin) for 30 min. Sections were incubated with primary antibodies as follows: mouse anti-mCherry antibody (1:1000) + guinea pig anti-VGluT1 antibody (1:500) + rabbit anti-VIP (Vasoactive Intestinal Peptide) antibody (1:1000, 20077, ImmunoStar, Wisconsin). All primary antibodies were diluted with 1% normal goat serum (NGS), 4% BSA in PBS supplemented with 0.02% saponin and incubations were for 24 h at 4°C. Sections were rinsed and incubated overnight at 4°C in the corresponding secondary antibodies: biotinylated anti-mouse (mCherry detection) + anti-guinea pig-IgG Fab′ fragment coupled to 1.4-nm gold (VGluT1 detection, 1:100, 2055-1ML, Nanoprobes Inc., Yaphank, NY) + anti-rabbit-IgG coupled to 1.4-nm gold (VIP detection, 1:100, 2004-1ML, Nanoprobes Inc.). Sections were rinsed in PBS and then incubated in avidin-biotinylated horseradish peroxidase complex in PBS for 2 h at room temperature. Sections were rinsed in PBS and postfixed with 1.5% glutaraldehyde at room temperature for 10 min. Sections were rinsed again in PBS and in double-distilled water, followed by silver enhancement of the gold particles with the Nanoprobe Silver Kit (2012, Nanoprobes Inc.) for 7 min at room temperature. Next, peroxidase activity was detected with 0.025% 3,3’-diaminobenzidine (DAB) and 0.003% H_2_O_2_ in PBS for 5-10 min. Sections were rinsed with PBS and fixed with 0.5% osmium tetroxide in PBS for 25 min, washed in PBS followed by double distilled water, and contrasted in freshly prepared 1% uranyl acetate for 35 min. Sections were dehydrated through a series of graded alcohols and propylene oxide, and flat embedded in Durcupan ACM epoxy resin (14040, Electron Microscopy Sciences, Hatfield, PA). Resin-embedded sections were polymerized at 60°C for 2 days, and sections of 60 nm were cut from the outer surface of the tissue with an ultramicrotome UC7 (Leica Microsystems, Deerfield, IL) using a diamond knife (Diatome, Hatfield, PA). The sections were collected on formvar-coated single slot grids and counterstained with Reynolds lead citrate to be examined and photographed using a Tecnai G2 12 transmission electron microscope (Fei Company, Hillsboro, OR) equipped with the OneView digital micrograph camera (Gatan, Pleasanton, CA).

#### Ultrastructural analysis.

Serial ultrathin sections of the mPFC from 12 mice (6 WT and 6 *Df(16)A*^+/−^) were analyzed. Synaptic contacts were classified according to their morphology and immunolabel and imaged at 6,800-13,000x. The morphological criteria used for identification and classification of cellular components or type of synapse observed in these thin sections were as previously described ^73^. In the serial sections, a terminal containing more than three immunogold particles was considered immuno-positive. Images were adjusted to match contrast and brightness by using Adobe Photoshop (Adobe Systems). This experiment was successfully repeated three times. Electron microscopy quantification was blinded.

#### Histology.

Following experiments, mice were anesthetized with isoflurane (5% in oxygen, v/v) and transcardially perfused with 10mL phosphate buffered saline (PBS) followed by 10mL of 4% paraformaldehyde (PFA, FD Neurotechnologies). Brains were post-fixed for 24-48h in 4% PFA and transferred to PBS. Brains were cut into 50-μm slices on a vibratome (Camden Instruments 7000). Slices were mounted with DAPI Fluoromount-G (Southern Biotech) on glass slides (MS10UW, ThorLabs) and coverslipped (CG15KH, ThorLabs) to visualize optic fiber placements and viral expression using a custom epifluorescence (Leica), confocal (LSM800, Zeiss), or slide-scanning (Axioscan 7, Zeiss) microscope. Fiber placements and viral expression were mapped onto templates of a stereotaxic mouse brain atlas (Franklin & Paxinos, 2007).

#### Statistical analyses.

Statistical analyses were performed using GraphPad Prism and custom Python/R scripts. For the opto-photometry and SWM experiments, 2- or 3-way repeated measures ANOVA were used to assess a combination of between-subjects (e.g., stimulation) and within-subjects factors (e.g., day). In rare instances where a small fraction of datapoints were randomly missing, mixed-effects analyses were conducted in Prism. Significance (p<0.05) of posthoc comparisons was determined after applying Tukey’s/Dunnet’s multiple comparisons tests. Pairwise comparisons (e.g., NS vs. HFS oEPSCs) were conducted using Mann Whitney and unpaired t tests, depending on sample size and distribution normality. Kolmogorov-Smirnov tests were performed on cumulative distributions of days to criterion data. Pearson correlations were used to quantify relationships between Ca^2+^ signals and behavioral performance. The linear mixed-effects model function in R, “lmer”, was used to assess within-session changes in stimulation-evoked Ca^2+^ responses (fixed effect: trial; random intercept and slope for each mouse). Unless stated otherwise, error bars and shaded bands represent standard error of the mean.

### Functional linear mixed modeling.

Functional linear mixed modeling (FLMM) was performed using the fastFMM toolkit ^48,49^, implemented in R. Individual task event-level Ca^2+^ signals spanning 10-sec windows were analyzed using five FLMMs:

The first (1) modeled Ca^2+^ signals as a function of the fixed effect of Stimulation with a random intercept for each mouse (photometry ~ stim + (1 | mouse_id)). Specifically, for animal *i*, on event *e*, we modeled the Ca^2+^ signals at time *t* as:

$${\text{Photometry}}_{i,e}(t)\;=\beta_{0}(t)\;+\gamma_{0,i}(t)\;+\beta_{1}(t){\text{Stimulation}}_{i}+\epsilon_{i,e}(t)$$

where $\epsilon_{i,e}(t)∼N(0,\sigma^{2}(t))$ and $\gamma_{0,i}(t)∼N(0,\sigma^{2}\gamma (t))$. We denoted Stimulation_*i*_ = 0 for NS mice and Stimulation_*i*_ = 1 for HFS mice. This model was fit to datasets of VIP or SST interneuron Ca^2+^ signals from either correct trials (Figure 6B) or incorrect trials (data not shown).

The second (2) modeled Ca^2+^ signals as a function of the fixed effect of Outcome with a random slope and intercept for each mouse (photometry ~ outcome + (outcome | mouse_id)). Specifically, for animal *i*, on event *e*, we modeled the Ca^2+^ signals at time *t* as:

$${\text{Photometry}}_{i,e}(t)\;=\beta_{0}(t)\;+\gamma_{0,i}(t)\;+\;[\beta_{1}(t)\;+{\text{γ}}_{1,i}(t)]{\text{Outcome}}_{i,e}+\epsilon_{i,e}(t)$$

where $\epsilon_{i,e}(t)∼N(0,\sigma^{2}(t))$ and $\gamma_{i,e}(t)∼N(0,\Sigma \gamma (t))$. We denoted Outcome_*i,e*_ = 0 for Correct trials and Outcome_*i,e*_ = 1 for Incorrect trials. This model was fit to datasets of VIP or SST interneuron Ca^2+^ signals from either NS or HFS mice (Figure S5E).

The third (3) modeled Ca^2+^ signals as a function of two fixed effects (Training and Outcome) and their interaction with a random slope and intercept for Outcome for each mouse (photometry ~ training * outcome + (outcome | mouse_id)). Specifically, for animal *i*, on event *e*, we modeled the Ca^2+^ signals at time *t* as:

$${\text{Photometry}}_{i,e}(t)\;=\beta_{0}(t)\;+\gamma_{0,i}(t)\;+\beta_{1}(t){\text{Training}}_{i,e}+\;[\beta_{2}(t)\;+\gamma_{1,i}(t)]{\text{ Outcome}}_{i,e}+\beta_{3}(t){\text{Training}}_{i,e}\times {\text{ Outcome}}_{i,e}+\epsilon_{i,e}(t)$$

where $\epsilon_{i,e}(t)∼N(0,\sigma^{2}(t))$ and $\gamma_{i,e}(t)∼N(0,\Sigma \gamma (t))$. We analyzed Training_*i,e*_ as a continuous variable, where 0 corresponds to the first trial and 1 corresponds to the last trial of a given mouse. We denoted the binary variable Outcome_*i,e*_ = 0 for Correct trials and Outcome_*i,e*_ = 1 for Incorrect trials. This model was fit to a dataset of VIP interneuron Ca^2+^ signals from NS mice (Figure 7B, S6A).

The fourth (4) modeled Ca^2+^ signals as a function of two fixed effects (Training and Stimulation) and their interaction with a random intercept for each mouse (photometry ~ training * stim + (1 | mouse_id)). Specifically, for animal *i*, on event *e*, we modeled the Ca^2+^ signals at time *t* as:

$${\text{Photometry}}_{i,e}(t)\;=\beta_{0}(t)\;+\gamma_{0,i}(t)\;+\beta_{1}(t){\text{Training}}_{i}+\beta_{2}(t){\text{Stimulation}}_{i}+\epsilon_{i,e}(t)$$

where $\epsilon_{i,e}(t)∼N(0,\sigma^{2}(t))$ and $\gamma_{i,e}(t)∼N(0,\Sigma \gamma (t))$. We analyzed Training_*i*_ as a continuous variable, where 0 corresponds to the first trial and 1 corresponds to the last trial of a given mouse. We denoted Stimulation_*i*_ = 0 for WT mice and Stimulation_*i*_ = 1 for HFS mice. This model was fit to a dataset of VIP interneuron Ca^2+^ signals from NS and HFS mice (Figure 7D, S6A, S7).

The fifth (5) model was identical to the fourth, except that we replaced Stimulation_*i*_ with Genotype_*i*_ (photometry ~ training * genotype + (1 | mouse_id)), and denoted Genotype_*i*_ = 0 for WT (NS) mice and Genotype_*i*_ = 1 for *Df(16)A*^+/−^ mice. This model was fit to a dataset of VIP interneuron Ca^2+^ signals from NS and *Df(16)A*^+/−^ mice (Figure 7G, S6D).

FLMM was also used to quantify differences in the Ca^2+^-velocity cross-correlations of SST and VIP interneuron recordings from WT, NS mice. Individual trial-level cross-correlations spanning ±5 sec lead/lag timepoints were modeled as a function of the fixed effect of CellType with a random intercept for each mouse (cc ~ cell_type + (1 | mouse_id)). Specifically, for animal *i*, on trial *j*, we modeled the cross-correlations at time *t* as:

$${\text{Photometry}}_{i,j}(t)\;=\beta_{0}(t)\;+\gamma_{0,i}(t)\;+\beta_{1}(t){\text{CellType}}_{i}+\epsilon_{i,j}(t)$$

where $\epsilon_{i,j}(t)∼N(0,\sigma^{2}(t))$ and $\gamma_{0,i}(t)∼N(0,\sigma^{2}\gamma (t))$. We denoted CellType_*i*_ = 0 for SST-velocity cross-correlations and CellType_*i*_ = 1 for VIP-velocity cross-correlations. This model was fit to a dataset of Ca^2+^-velocity cross-correlations from NS mice (Figure S5G).

Prior to selection, models were tested using different random effects structures; models that successfully converged and yielded lower AIC/BIC values were selected. FLMM generates Pointwise and Joint 95% confidence intervals (CIs) around the resulting functional coefficients. Timepoints at which Pointwise 95% CIs do not contain Y = 0 indicate individual timepoints of statistical significance, without correction for multiple comparisons. Joint 95% CIs, generated by exploiting the correlation between neighboring timepoints, are adjusted for multiple comparisons; thus, time intervals during which Joint 95% CIs do not contain 0 are interpreted as statistically significant.

### Computational Modeling.

An integrate-and-fire computational model was created to simulate how a mPFC neural circuit composed of pyramidal neurons and three classes of interneurons (VIP, SST, and PV) might respond to stimulation of vHPC inputs. The model circuit consisted of four VIP cells, six SST cells, six PV cells, and twenty pyramidal cells. Each cell type received inputs from the vHPC, mPFC pyramidal cells, and a subset of mPFC interneuron types. Synaptic weights were informed by present data (e.g., Figures 3E,F) and primary literature^26,44,74,75^. Synaptic weights also reflected the canonical view of VIP cells as providing ‘disinhibitory’ inhibition onto other interneurons (predominantly SST interneurons), SST cells as providing ‘feedback’ inhibition, and PV cells as providing ‘feedforward’ inhibition ^31–33^.

The synaptic weights used in the model were as follows:

**Table:** 

From:	To:			
	VIP	SST	PV	PYR
vHPC	1.6**	1	1.6	1
VIP	0	1	0.05	0
SST	1	0	0.75	0.75
PV	0	0	2.25	2
PYR	0.8	8	0.8	0.25*

* Not all pyramidal cells synapsed onto all other pyramidal cells; the connection probability between any two pyramidal cells was 0.2.

** This value was later modified in our exploration of whether decreased vHPC inputs to VIP cells might plausibly reproduce the altered dynamics we observed experimentally (e.g., Figure 1).

Network activity was simulated over the course of a 10-sec period, with vHPC input stimulation occurring from time = 2 to 3 sec. Each neuron in each cell class began with a membrane voltage of 0, and at every subsequent timestep (dt = 0.1 ms) the new membrane voltage of each neuron was calculated based on the current activity levels of each of its inputs, multiplied by the corresponding synaptic weight. Specifically, the following equation was used to estimate the membrane potential of neuron *i* from interneuron cell class *x* at timepoint *t*:

$$v_{x,i}\left(t\right)=v_{x,i}\left(t-1\right)*vdecay_{x}\;\;+dt*\left(-w_{SST,x}*out_{SST}\left(t-1\right)-w_{VIP,x}*out_{VIP}\left(t-1\right)-w_{PV,x}*out_{PV}(t-1\right)\;\;+w_{PYR,x}\left(out_{PYR-AMPA}\left(t-1\right)+0.3out_{PYR-NMDA}\left(t-1\right)\right)\;\;+w_{vHPC,x}\left(out_{vHPC-AMPA}\left(t-1\right)+0.3out_{vHPC-NMDA}(t-1\right)\;\;+out_{vHPC-noise}\left(t-1\right))+noise_{x,i}\left(t-1\right))$$

where *w_A,x_* is the synaptic weight of neurons from cell class *A* onto neurons in cell class *x*; *out_A_*(*t*) represents the synaptic output of cell class *A* at time *t*; *noise_x,i_*(*t*) is the random noise input which differs between individual neurons from cell class *x*; and *vdecay_x_ = e^−dt/τ_x_^* describes the decay of a neuron’s membrane potential back to its resting potential over time, with a time constant τ = 10 ms for all cell types. The amplitudes of NMDA outputs were scaled by a factor of 0.3, as informed by the slice electrophysiology data (Figure 3F,H). Whenever a given neuron’s membrane potential crossed a threshold of 10, it was said to have fired a spike, and the membrane potential was reset to −10 at the next timestep.

For neurons from the pyramidal cell class, this equation was modified to:

$$v_{x,i}\left(t\right)=v_{x,i}\left(t-1\right)*vdecay_{x}\;\;+dt*\left(-w_{SST,x}*out_{SST}\left(t-1\right)-w_{VIP,x}*out_{VIP}\left(t-1\right)-w_{PV,x}*out_{PV}(t-1\right)\;\;+M_{PYR}w_{PYR,x}\left(out2_{PYR-AMPA}\left(t-1\right)+0.3out2_{PYR-NMDA}\left(t-1\right)\right)\;\;+w_{vHPC,x}\left(out_{vHPC-AMPA}\left(t-1\right)+0.3out_{vHPC-NMDA}(t-1\right)\;\;+out_{vHPC-noise}\left(t-1\right))+noise_{x,i}\left(t-1\right))$$

where *M_PYR_* is a 20x20 connectivity matrix between the twenty pyramidal cells (connection probability between any two cells = 0.2), and *out2* is a 20x1 matrix describing the synaptic output of individual pyramidal cells.

To determine the synaptic output from a given mPFC cell class (*out_PV_, out_SST_, out_VIP_, out_PYR–AMPA_*, or *out_PYR–NMDA_*) at a given timepoint *t*, the following steps were performed. At t = 0, all mPFC synaptic outputs were set to 0. At each subsequent timestep, the new membrane voltages were calculated using the equation above. Next, the proportion of cells from each cell class whose membrane potential had crossed the firing threshold (threshold = 10) was determined; this proportion was called *P_x_*. The synaptic output of a cell class *x* was then calculated as:

$$out_{x}\left(t\right)=out_{x}\left(t-1\right)*syndecay_{x}+P_{x}\left(t\right)$$

where the time constants τ for synaptic decay were 20 ms for all interneuron classes, 10 ms for pyramidal AMPA currents, and 80 ms for pyramidal NMDA currents. The same equation was used to calculate each element of the 20x1 *out2* matrix, with P_x_ simply being 1 if that pyramidal cell had fired and 0 otherwise.

To determine the vHPC input synaptic output at a given timepoint *t*, a raster of vHPC input spike events was created. From time = 0 to 2 sec, the rate of vHPC input firing was 0 Hz. From time = 2.0001 to 3 sec (i.e., during vHPC input ‘stimulation’), the expected rate of vHPC input firing was 100 Hz, meaning that the expected number of events within 1 sec was 100 (although the exact timing of the events was random). After time = 3 sec, the vHPC input event rate gradually decayed down from 100 Hz, with a time constant τ of 1000 ms. To determine the AMPA component of the vHPC input synaptic output *out_vHPC–AMPA_* (*t*), the event raster was smoothed by allowing each event (of amplitude 1) to decay over 40 timesteps with a time constant τ of 8 ms; the value of the smoothed trace at any timepoint *t* could then be found. To determine the NMDA component of the vHPC input synaptic output *out_vHPC–NMDA_*(*t*), the event raster was smoothed by allowing each event to decay over 400 timesteps using a time constant τ of 80 ms. Finally, all cell types also received additional noise input from the vHPC input, *out_vHPC–noise_*(*t*), representing background vHPC input that occurs even in the absence of vHPC input stimulation. Noise events occurred at an expected rate of 100 Hz throughout the entire 10-sec duration of the simulation, had an event amplitude of 1, and decayed with a time constant τ of 8 ms over 40 timesteps.

Each neuron also received neuron-specific noise, *noise_x,i_*(*t*). Random noise events for each neuron occurred at an expected rate of 100 events/s, had an amplitude of 1 (except for PV cells, where the amplitude was scaled to 1.2), and decayed over 40 timesteps with a time constant τ of 8 ms.

During each run of the model, the total number of spikes from each cell class at each timepoint was recorded. After the run, these traces of summed spike counts were converted to photometry-like activity traces by smoothing with a Gaussian filter with sigma = 25 msec. The final activity traces from each simulation were the average of five runs.

To test the hypothesis that decreased vHPC input onto VIP interneurons might explain the observed dynamics (Figure 1), the value of *w_vHPC ,VIP_* was systematically decreased from its baseline value of 1.6. Ten simulations were run for each synaptic weight value, with each simulation consisting of the average of five runs of the model. Means and SEMs were calculated using the output from the ten simulations (n = 10). To look at vHPC input stimulation-‘evoked’ activity, baseline activity for each cell type was calculated as the mean of the activity traces from time = 0.1 to 2 sec and was subtracted from the overall activity trace.

### RESOURCE AVAILABILITY

#### Lead Contact

Further information and requests for resources and reagents should be directed to and will be fulfilled by the lead contact, David Kupferschmidt (david.kupferschmidt@nih.gov).

#### Materials availability

This study did not generate new unique reagents.

#### Data and code availability

Code associated with this paper is found at https://doi.org/10.5281/zenodo.16277905. Data associated with this paper will be shared by the lead contact upon request.

## Supplementary Material

Supplement 1

## HIGHLIGHTS

- Repeated stimulation of vHPC inputs to mPFC persistently depresses VIP interneuron activity and enhances SST interneuron activity in mice
- Blunted monosynaptic drive onto mPFC VIP interneurons by repeated vHPC input stimulation plausibly reproduces *in vivo* activity changes
- Stimulated mice show heightened VIP interneuron activity during the delay epoch in early SWM task training that correlates with poorer training performance
- Mice modeling the 22q11.2 deletion syndrome with known SWM task learning deficits mirror this aberrant VIP interneuron activity profile and show reduced vHPC input targeting of VIP interneurons
