# Supplementary material for: Hippocampal input-driven plasticity of prefrontal interneurons reveals a circuit basis for impaired spatial working memory
Source: bioRxiv. 2025 Jul 24:2025.07.21.665987. Preprint. [Version 1] doi: 10.1101/2025.07.21.665987 (PMC12330631; doi:10.1101/2025.07.21.665987)
Supplement: Supplement 1 [file media-1.pdf]

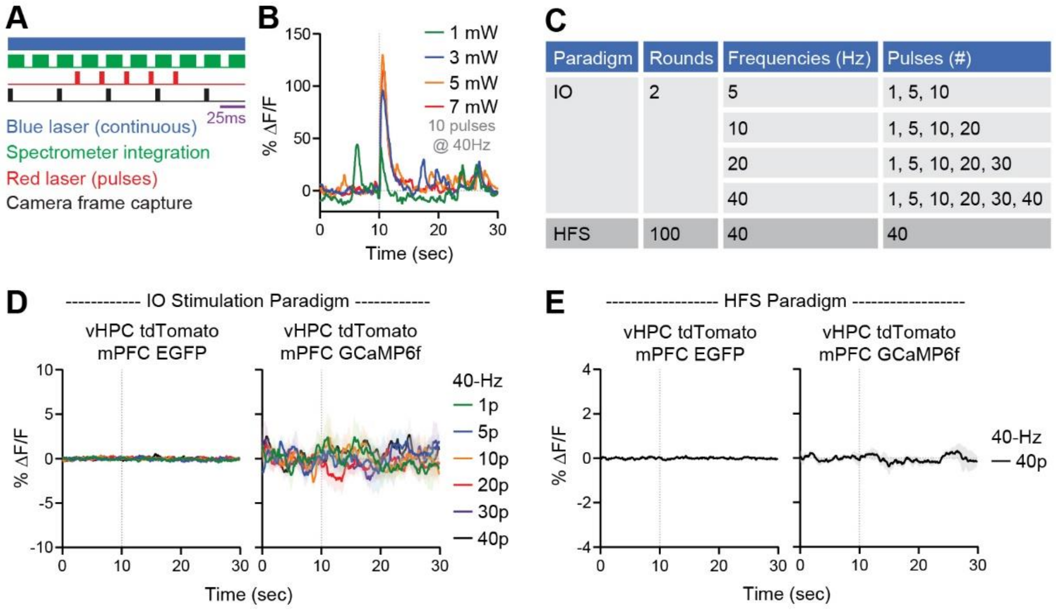

Supplemental Figure 1. Parameterizing the opto-photometry system and paradigm and artifact testing in SST::Cre mice.

(**A**) Schematic illustrating all-optical approach to simultaneously stimulate vHPC inputs and record Ca^{2+} activity in mPFC neurons in behaving mice. (**B**) Stimulation-evoked Ca^{2+} responses from a representative pilot SST::Cre mouse to different red laser outputs (i.e., different vHPC input stimulation intensities). (**C**) Table description of rounds, frequencies (Hz), and pulse numbers of redlight stimulation delivered during IO and HFS sessions. (**D**) Average SST interneuron photometry recordings during IO stimulation (various pulse numbers at 40 Hz) in mice expressing control fluorophores. Left: Mice (n=4) expressing tdTomato in vHPC and EGFP in mPFC SST interneurons. Right: Mice (n=4) expressing tdTomato in vHPC and GCaMP6f in mPFC SST-INs. (**E**) Average SST interneuron photometry recordings during HFS stimulation in mice expressing control fluorophores. Left: Mice (n=4) expressing tdTomato in vHPC and EGFP in mPFC SST interneurons. Right: Mice (n=4) expressing tdTomato in vHPC and GCaMP6f in mPFC SST interneurons.

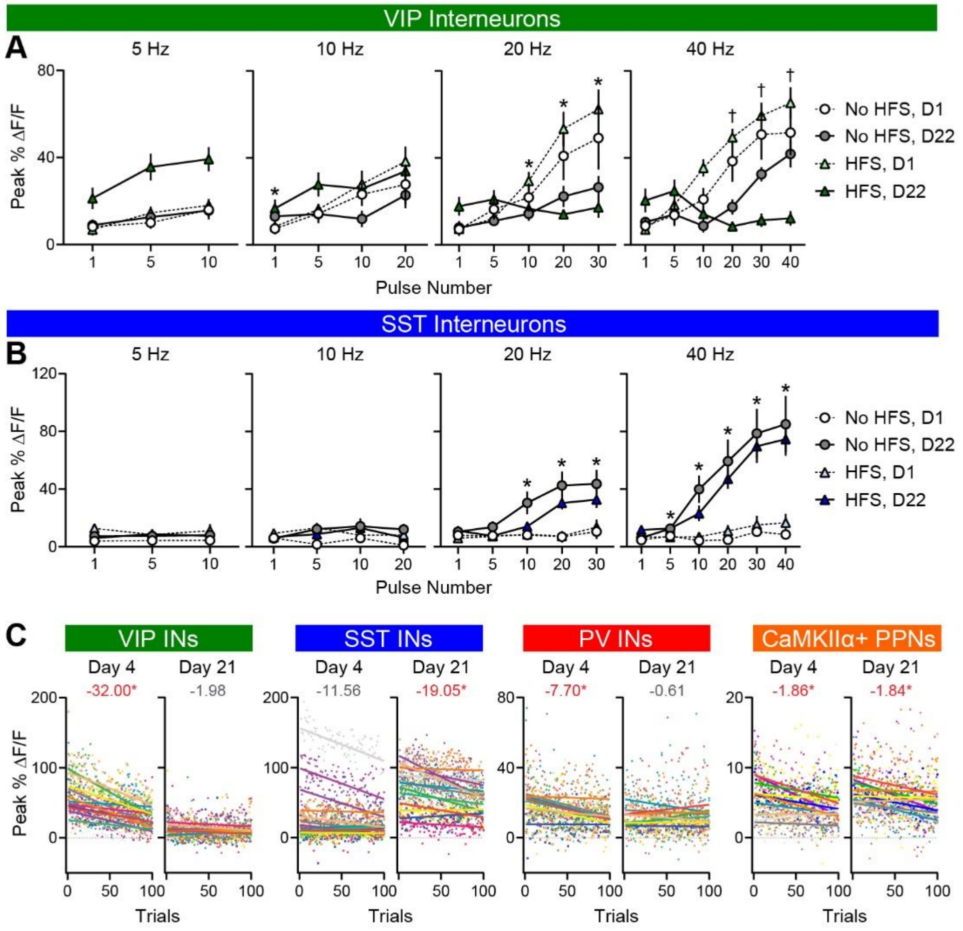

Supplemental Figure 2. vHPC input stimulation response curves and within-session effects in mPFC neuron populations.

(**A**) Average peak stimulation-evoked Ca^{2+} activity in VIP interneurons at varying frequencies and pulse numbers in No HFS and HFS mice on IO Days 1 and 22. 3-way ANOVA, 10 Hz: Pulse Number x Day interaction: $F(2.735, 57.43)=4.000$, $p<0.05$; *$p<0.05$, Day 1 vs. Day 22. 20 Hz: Pulse Number x Day interaction: $F(1.364, 28.64)=20.63$, $p<0.0001$; Pulse Number x Stimulation x Day interaction: $F(4, 84)=4.622$, $p<0.005$; *$p<0.05$, Day 1 vs. Day 22. 40 Hz: Pulse Number x Stimulation interaction: $F(5, 105)=3.529$, $p<0.01$; Pulse Number x Day interaction: $F(2.592, 54.42)=19.51$, $p<0.0001$; Stimulation x Day interaction: $F(1, 21)=4.613$, $p<0.05$; Pulse Number x Stimulation x Day interaction: $F(5, 105)=6.475$, $p<0.0001$; †$p<0.005$, HFS Day 1 vs. HFS Day 22; n=11-12. (**B**) Average peak stimulation-evoked Ca^{2+} activity in SST interneurons at varying frequencies and pulse numbers in No HFS and HFS mice on IO Days 1 and 22. 3-way ANOVA, 20 Hz: Stimulation x Day interaction: $F(1, 23)=4.749$, $p<0.05$. 40 Hz: Main effect of Pulse Number: $F(2.573, 59.17)=15.99$, $p<0.0001$; Main effect of Day: $F(1, 23)=21.57$, $p<0.0001$; Pulse Number x Day interaction: $F(1.759, 40.46)=10.79$, $p<0.0005$; *$p<0.05$, Day 1 vs. Day 22; n=12-13. (**C**) Peak stimulation-evoked Ca^{2+} activity in VIP, SST, or PV interneurons and CaMKIIα+ putative pyramidal neurons for all 100 trials on HFS Days 4 and 21. Linear Mixed Models; Fixed effect of Trial reported above each panel; grey text: $p>0.05$; red text and *: $p<0.05$.

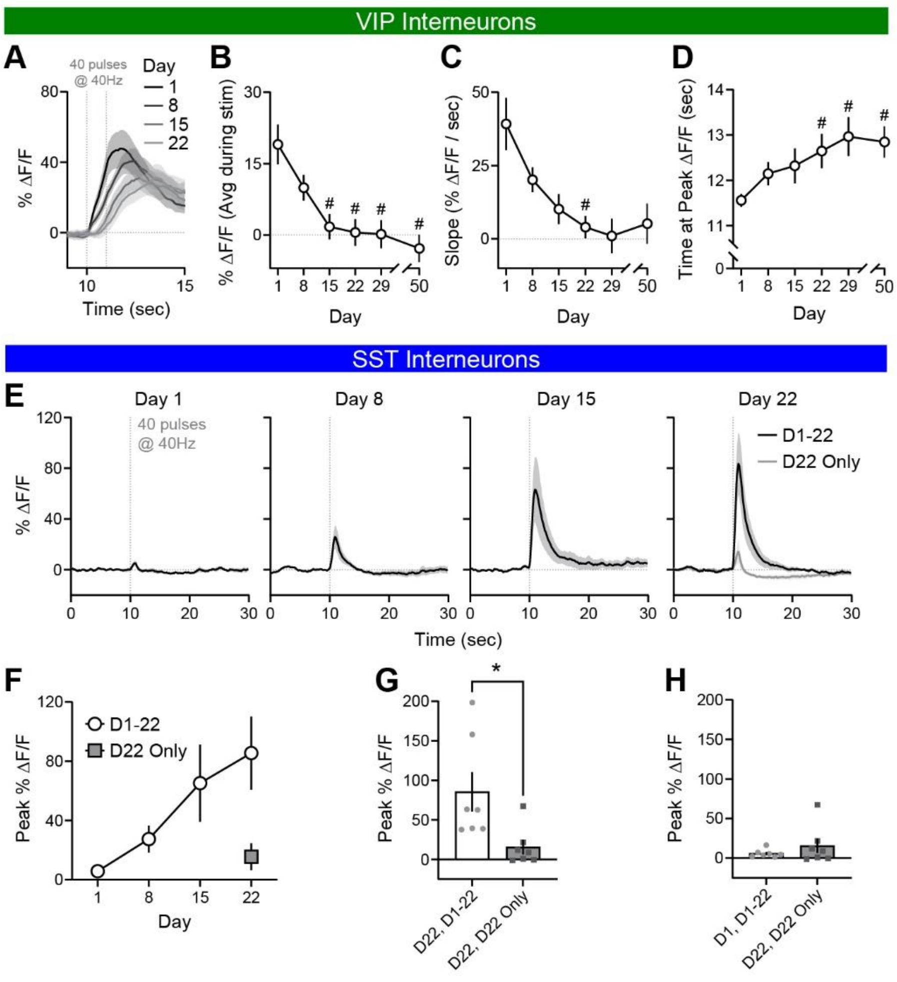

Supplemental Figure 3. Characterizing VIP and SST interneuron Ca^{2+} responses to input-output stimulation of vHPC inputs to mPFC.

(**A**) Average stimulation-evoked Ca^{2+} responses of mPFC VIP interneurons on Days 1, 8, 15, and 22 in No HFS mice (n=11). Stimulation (40 pulses at 40 Hz) initiated at 10-sec timepoint. (**B**) Average stimulation-evoked Ca^{2+} responses during 1-sec stimulation (40 pulses at 40 Hz) on Days 1, 8, 15, 22, 29, and 50. Repeated measures ANOVA, Main effect of Day: F(2.309, 23.09)=8.394, p<0.005; #p<0.05, different from Day 1; n=11. (**C**) Slope of average stimulation-evoked Ca^{2+} responses across 1-sec stimulation on Days 1, 8, 15, 22, 29, and 50. Repeated measures ANOVA, Main effect of Day: F(1.455, 14.55)=6.582, p<0.05; #p<0.05, different from Day 1; n=11. (**D**) Time at peak Ca^{2+} response to stimulation on Days 1, 8, 15, 22, 29, and 50. Repeated measures ANOVA, Main effect of Day: F(3.321, 33.21)=4.145, p<0.05; #p<0.05, different from Day 1; n=11. (**E**) Average stimulation-evoked Ca^{2+} responses of mPFC SST interneurons on Days 1, 8, 15, and 22 for D1-22 and D22 Only groups (n=7,7). Neither group received HFS during intervening days. (**F**) Average peak stimulation-evoked Ca^{2+} responses of mPFC SST interneurons in D1-22 and D22 Only groups. (**G**) Average peak stimulation-evoked Ca^{2+} responses on Day 22 in D1-22 and D22 Only groups. *p<0.05, U=5, Mann-Whitney test; n=7,7. (**H**) Average peak stimulation-evoked Ca^{2+} responses of D1-22 on Day 1 and D22 Only mice on Day 22.

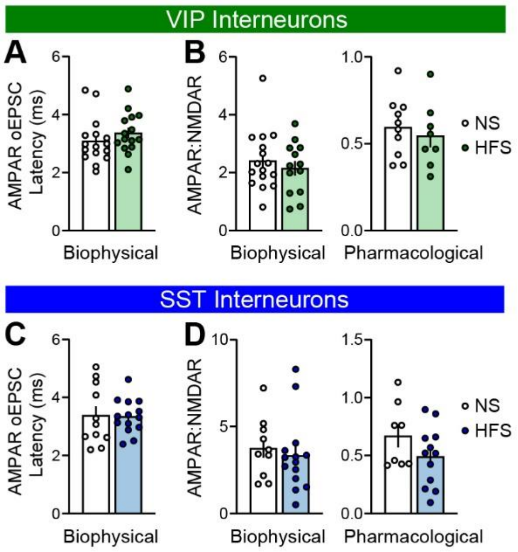

Supplemental Figure 4. Additional parameters from brain slice whole-cell electrophysiology of monosynaptic connectivity between vHPC inputs and mPFC interneurons.

(**A**) Latency of biophysically isolated AMPAR-mediated monosynaptic oEPSCs in VIP interneurons of NS and HFS mice (n=15-16 cells). (**B**) Biophysically (left) and pharmacologically (right) isolated AMPAR:NMDAR ratios in VIP interneurons of NS and HFS mice (n=8-16 cells). (**C**) Latency of biophysically isolated AMPAR-mediated monosynaptic oEPSCs in SST interneurons of NS and HFS mice (n=11-14 cells). (**D**) Biophysically (left) and pharmacologically (right) isolated AMPAR:NMDAR ratios in SST interneurons of NS and HFS (n=8-14 cells).

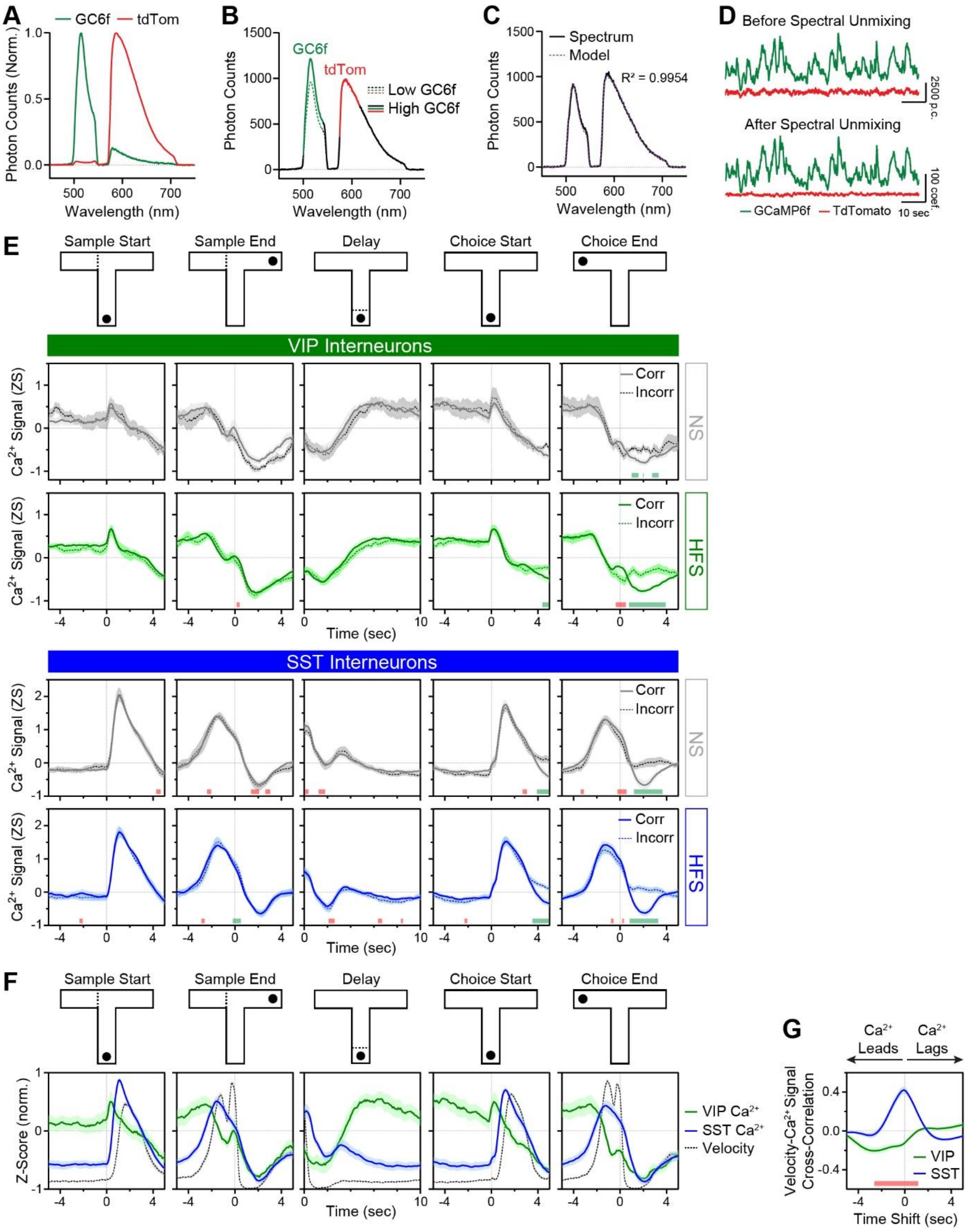

Supplemental Figure 5. Spectral unmixing and task-related VIP and SST interneuron Ca^{2+} and velocity measures.

(**A**) Normalized photon counts of GCaMP6f and tdTomato reference spectra recorded from mice solely expressing GCaMP6f or tdTomato. (**B**) Photon counts spanning the mixed GCaMP6f-tdTomato spectrum of a representative SST::Cre mouse at two experimental timepoints. The timepoints correspond to high- (solid lines) and low-points (dashed lines) in the GCaMP6f timeseries to show the dynamics changes to the GCaMP portion of the spectrum and relative stability of the tdTomato portion. (**C**) Photon counts of a single representative measured spectrum overlayed with its modeled spectrum generated by the linear unmixing algorithm. (**D**) Representative GCaMP6f and tdTomato traces showing photon counts (before spectral unmixing) and coefficient (after spectral unmixing) of the two signals across time. (**E**) Average Z-scored Ca^{2+} signals of VIP and SST interneurons from NS and HFS mice across all training days and aligned to discrete SWM task epochs during correct and incorrect trials. n=10-16. Green and red bars denote timepoints of significant enhancement and reduction in incorrect relative to correct trials, respectively, using functional linear mixed modelling. (**F**) Average normalized Ca^{2+} activity of VIP (green) and SST interneurons (blue), and average normalized mouse velocity (gray dashed, combined VIP and SST interneuron mice) aligned to discrete task epochs of the SWM task. Data are normalized such that Z-scored Ca^{2+} signals from individual trials ranged from –1 to 1. Data are from correct trials performed by NS mice only. n=10-14 for Ca^{2+}, n=24 for velocity. (**G**) Average cross-correlation of velocity and VIP (green) and SST interneurons (blue) or Ca^{2+} signals across whole trials of the SWM task. Red bar denotes timepoints of significant difference between VIP and SST interneuron velocity-Ca^{2+} cross-correlations using functional linear mixed modeling. n=10-14.

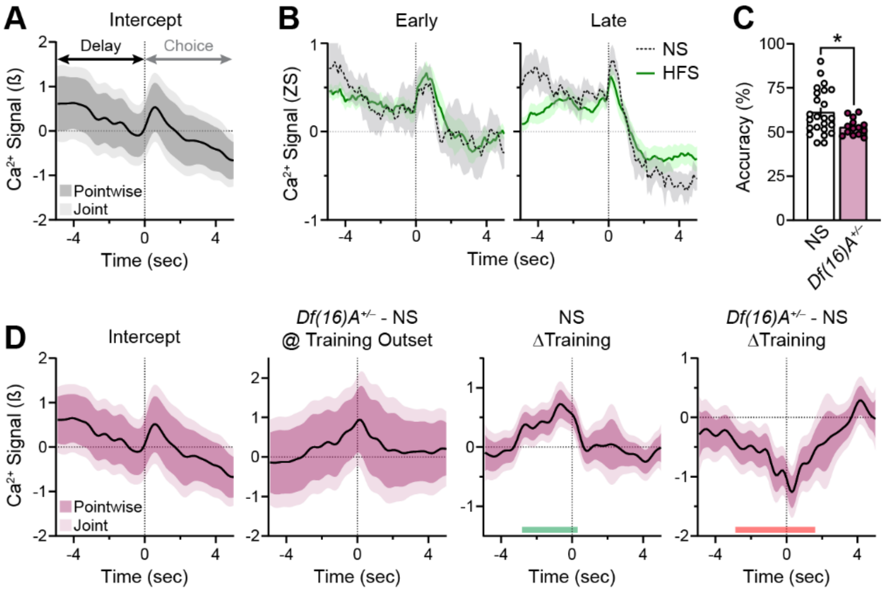

Supplemental Figure 6. Additional measures of delay epoch-related VIP interneuron Ca^{2+} activity and their modulation by prior vHPC input stimulation and *Df(16)A*$^{+/-}$ mutation.

(**A**) Functional intercept estimates of the Outcome, Training, and interaction between Outcome and Training covariates effects on trial-level VIP interneuron Ca^{2+} signals during the Delay-to-Choice transition (See Figure 7B). (**B**) Average Z-scored Ca^{2+} signals of VIP interneurons during the Delay-to-Choice transition for incorrect trials in NS and HFS mice in Early and Late stages of training. (**C**) Average accuracy (%) across training in NS and *Df(16)A*$^{+/-}$ mice. * unpaired t-test: $t(36)=2.36$, $p<0.05$; n=24,14. (**D**) Functional intercept estimates and coefficient estimates of the Genotype, Training, and interaction between Training and Genotype covariates effects on trial-level VIP interneuron Ca^{2+} signals during the Delay-to-Choice transition (See Figure 7G). Green and red bars denote timepoints of significant enhancement and reduction, respectively.

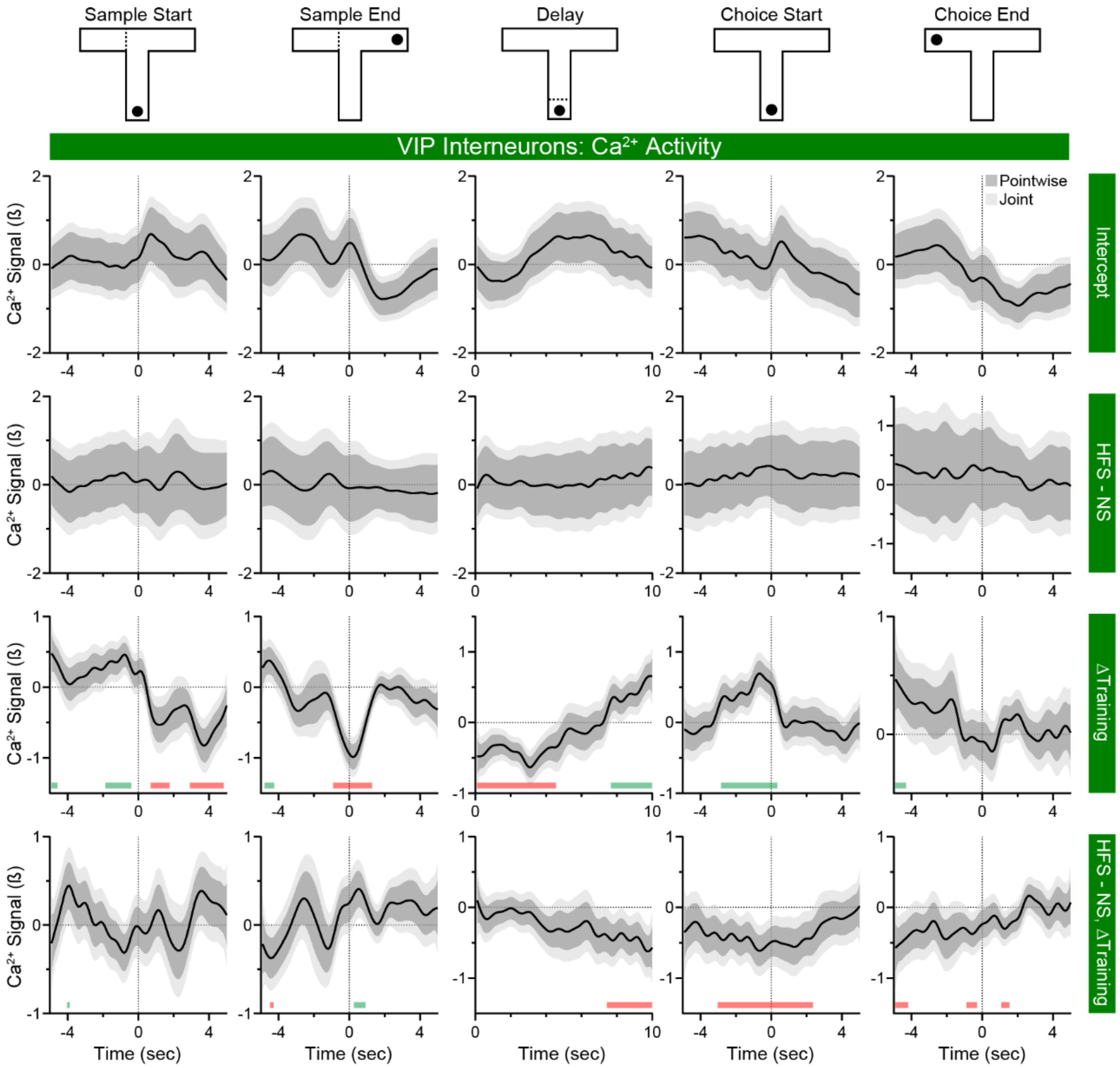

Supplemental Figure 7. Functional linear mixed-effects modeling of Stimulation, Training, and Stimulation x Training interactions in SWM task epochs for VIP interneuron Ca^{2+} data.

Functional intercept estimates and coefficient estimates of the Stimulation, Training, and the interaction between Stimulation and Training covariate effects on VIP interneuron Ca^{2+} signals around five SWM task events. Green and red bars denote timepoints of significant enhancement and reduction in coefficient values, respectively.

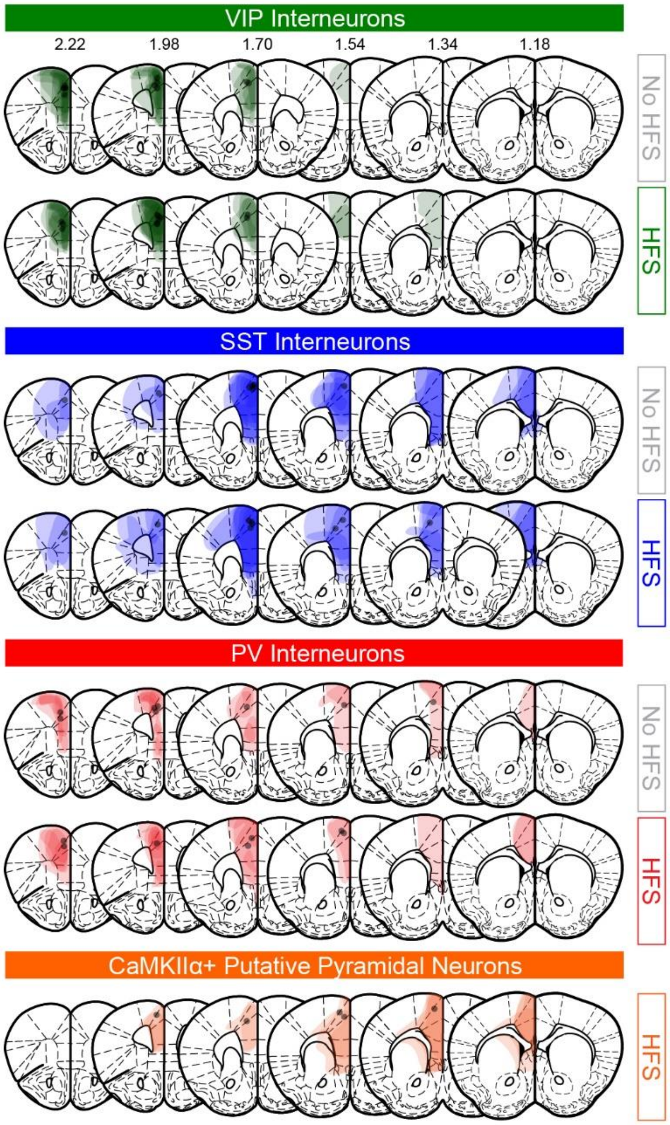

Supplemental Figure 8. Histology of GCaMP6f expression and optrode placement in mPFC for opto-photometry experiments.

AAV.Syn::FLEX.GCaMP6f or AAV.CaMKIIα::GCaMP6f expression in mPFC VIP, SST, or PV interneurons or CaMKIIα+ putative pyramidal neurons in No HFS and HFS mice. Gray circles indicate optical fiber placement.

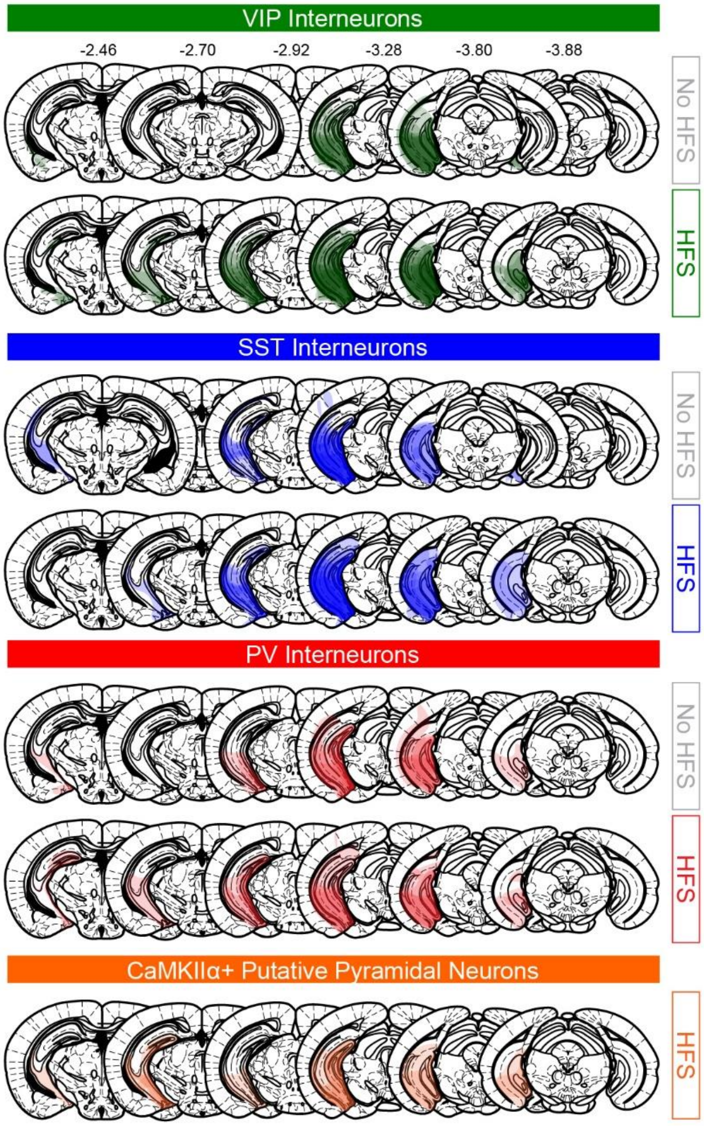

Supplemental Figure 9. Histology of ChrimsonR-tdTomato expression in vHPC for opto-photometry experiments.

AAV.Syn::ChrimsonR-tdTomato expression in vHPC in No HFS and HFS mice used for opto-photometry recordings from VIP, SST, or PV interneurons or CaMKIIα+ putative pyramidal neurons.

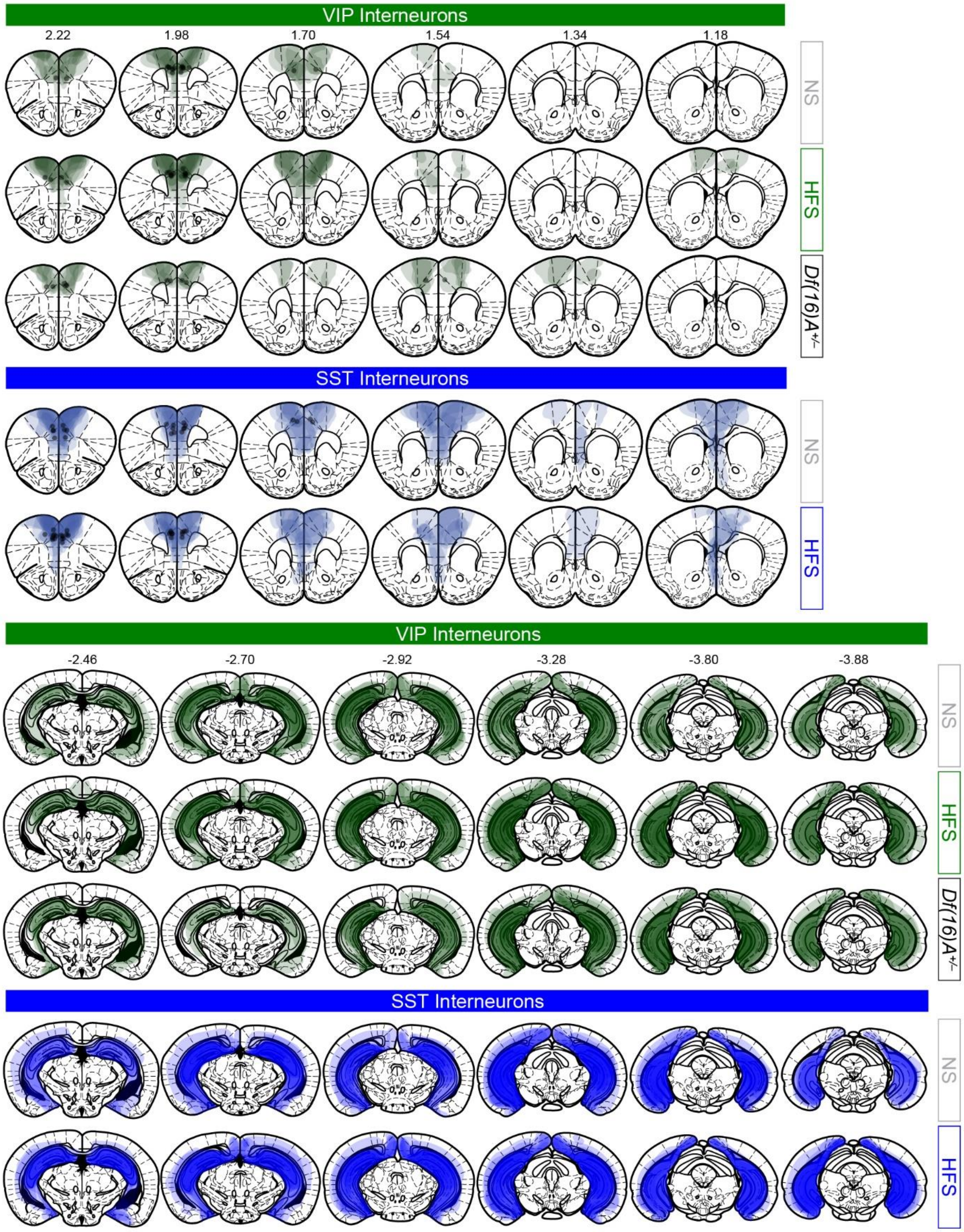

Supplemental Figure 10. Histology of ChrimsonR-tdTomato and GCaMP6f expression in mPFC and vHPC and optical fiber placement in mPFC for SWM experiments.

AAV.CaMKIIα::ChrimsonR-tdTomato expression in vHPC and AAV.Syn::FLEX.GCaMP6f in mPFC VIP and SST interneurons in NS, HFS, and *Df(16)A*$^{+/–}$ mice used for the SWM experiment. Gray circles indicate optical fiber placement.
